# Quantitative Surface Plasmon Interferometry via Upconversion Photoluminescence Mapping

**DOI:** 10.34133/2019/8304824

**Published:** 2019-09-15

**Authors:** Anxiang Yin, Hao Jing, Zhan Wu, Qiyuan He, Yiliu Wang, Zhaoyang Lin, Yuan Liu, Mengning Ding, Xu Xu, Zhe Fei, Jianhui Jiang, Yu Huang, Xiangfeng Duan

**Affiliations:** ^1^Department of Chemistry and Biochemistry, University of California, Los Angeles, California 90095, USA; ^2^School of Chemistry and Chemical Engineering, Beijing Institute of Technology, Beijing 100008, China; ^3^Department of Materials Science and Engineering, University of California, Los Angeles, California 90095, USA; ^4^State Key Laboratory for Chemo/Biosensing and Chemometrics, College of Chemistry and Chemical Engineering, Hunan University, Changsha 410082, China; ^5^Department of Physics & Astronomy, Iowa State University, Ames, Iowa 50011, USA; ^6^California NanoSystems Institute, University of California, Los Angeles, California 90095, USA

## Abstract

Direct far-field visualization and characterization of surface plasmon polaritons (SPPs) are of great importance for fundamental studies and technological applications. To probe the evanescently confined plasmon fields, one usually requires advanced near-field techniques, which is typically not applicable for real-time, high-throughput detecting or mapping of SPPs in complicated environments. Here, we report the utilization of rare-earth-doped nanoparticles to quantitatively upconvert invisible, evanescently confined SPPs into visible photoluminescence emissions for direct far-field visualization of SPPs in a complicated environment. The observed interference fringes between the SPPs and the coherent incident light at the metal surface provide a quantitative measurement of the SPP wavelength and the SPP propagating length and the local dielectric environments. It thus creates a new signaling pathway to sensitively transduce the local dielectric environment change into interference periodicity variation, enabling a new design of directly measurable, spectrometer-free optical rulers for rapid, ultrasensitive label-free detection of various biomolecules, including streptavidin and prostate-specific antigen, down to the femtomolar level.

## 1. Introduction

Surface plasmons are collective oscillations of electrons in conductors that confine electromagnetic field at the surface [1–6]. Direct visualization of surface plasmon polaritons (SPPs) and determination of SPP parameters are important and necessary for both fundamental studies and potential applications, including plasmonic waveguides [7, 8], antennas [9, 10], sensors [11–13], photovoltaics [14], and metamaterials [15–17]. However, the evanescently confined SPP fields cannot be directly observed with conventional far-field optical methods [3]. Near-field scanning optical microscopy (NSOM) is typically required for monitoring dispersion, propagation, and interference properties of SPPs [18–24]. However, NSOM protocols require flat and dry surfaces and cannot work on plasmonic resonators with biomolecule coverages or liquid environments [3]. The scanning probe method also prevents rapid, high-throughput mapping SPPs over a large area. An alternative approach to map SPPs is to use nanometer-scale photoluminescence (PL) probes that can be directly placed in the evanescent tail of the SPP field and excited by SPPs through spectrum coupling, in which the local field intensity is directly transduced into the emission intensity of the PL probes [3, 25].

Here, we report the utilization of rare-earth-doped (RE-doped) upconversion nanoparticles (UCNPs) [26, 27] as a unique class of optical probes that can quantitatively “upconvert” SPPs into visible PL emissions, rendering far-field PL images directly mapping the spatial dispersion of SPPs with high fidelity for the first time. The SPPs on Au patterns generated by a 980 or 808 nm laser can excite the UCNPs located in the evanescent tail of the SPP field. As a result, the spatial distribution of the local SPP field could be effectively converted into PL and mapped by UCNP PL emission intensity. This approach enables a highly convenient yet powerful method for probing the excitation, dispersion, propagation, and interference of SPPs in complicated environments. Notably, the super linear dependence of the upconversion emission intensity (*I*) on the excitation power (*P*) (*I* ∝ *P*^*n*^, where *n* is the number of photons involved in the energy-transfer-upconversion process for specific emissions) [28, 29] could further enhance the contrast of local field modulation in SPPs. Additionally, other advantages of UCNPs, including NIR excitation, widely tunable emissions, high stability, large anti-Stokes shift, and no photobleaching or blinking effects under high field intensity [26, 27, 30], make them ideal for the quantitative analysis of SPPs and their dependence on local environments. Significantly, the integration of SPP with UCPL creates a new signaling pathway to sensitively transduce local dielectric environment change into interference periodicity variation, enabling a new design of directly measurable, spectrometer-free optical rulers for ultrasensitive label-free detection of various biomolecules down to the fM level.

## 2. Results and Discussion

### 2.1. Far-Field Upconversion Visualization of Surface Plasmon Polaritons

**Figure 1:**
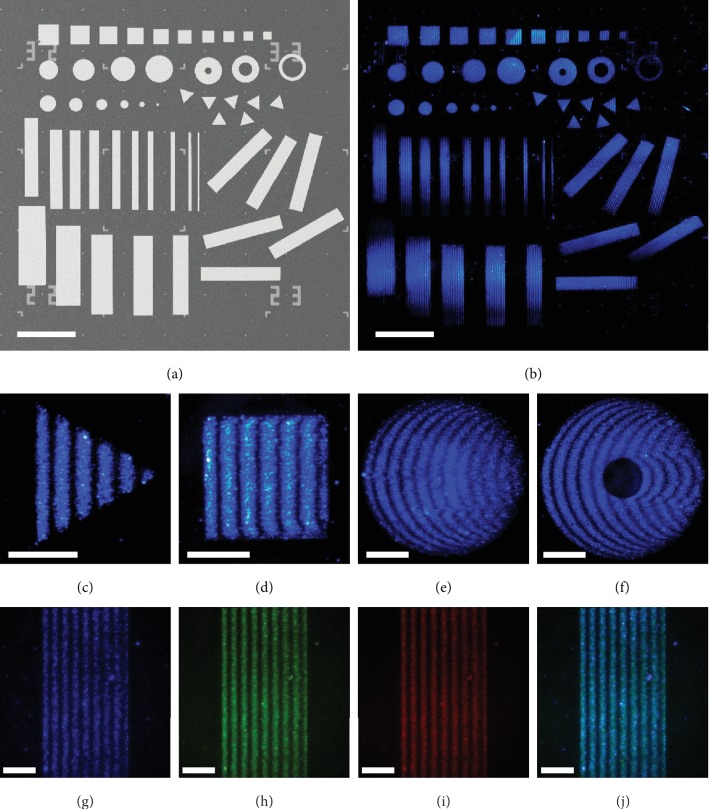
Direct mapping of surface plasmon polaritons (SPPs) by rare-earth-doped (RE-doped) upconversion nanoparticles (UCNPs). (a) SEM image of Au patterns (75 nm in thickness) on SiO_2_/Si substrate (SiO_2_ thickness: 300 nm). (b) Collaged real-color PL image showing interference fringes supported on Au-UCNP patterns with various morphologies (squares, rectangles, triangles, disks, and rings), sizes, and orientations. (c–f) PL images showing interference fringes supported on (c) Au triangle, (d) square, (e) disk, and (f) ring. (g–j) PL images acquired from (g) blue, (h) green, (i) red, and (j) merged channels showing interference fringes probed by UCNPs with different emission bands. The incident light for panel (b–j) is a TM mode 980 nm laser with an incident angle of 60°. Scale bar: (a, b) 200 *μ*m and (c–j) 25 *μ*m.

UCNPs were prepared through a solution process [28]. Typically, *β*–NaYF_4_:Yb,Tm and *β*–NaYF_4_:Yb,Er NPs can give a blue/red and green/red emission, respectively (Fig. [Supplementary-material supplementary-material-1] in the Electronic Supplementary Material (ESM)). The particle sizes are about 55 nm (Figs. [Supplementary-material supplementary-material-1] and [Supplementary-material supplementary-material-1] in the ESM) to ensure that they are well within the evanescent tail of the SPPs. Two-dimensional (2D) Au patterns with flat surfaces and specifically designed shapes were lithographically patterned on SiO_2_/Si substrates (Figure 1(a) and Fig. [Supplementary-material supplementary-material-1] in the ESM). UCNPs were subsequently spin-coated onto the Au patterns to form a randomly dispersed NP layer with a surface coverage of about 30% (Fig. [Supplementary-material supplementary-material-1] in the ESM). UCPL was excited by an obliquely shed NIR laser (*λ*_0_ = 980 nm, TM mode) and directly imaged by an optical microscope with a CCD camera (Fig. [Supplementary-material supplementary-material-1] in the ESM).

Systematically dispersed fringes can be observed on all Au patterns with various shapes (Figure 1). All fringes are dispersed periodically along the *x*-direction (horizontal direction), which is also the propagation direction of SPPs. Each stripe mimics the shape of the front edge of the respective Au pattern (Figures 1(c)–1(f)). For Au squares or triangles with a straight edge that is perpendicular to the incident light, the fringes show periodic vertical stripes that are parallel to the front edge (Figures 1(c) and 1(d)). For Au disks or rings, the stripes of the fringes follow exactly the curvature of the front edges (Figures 1(e) and 1(f)). Fringes can be also visualized in different PL color channels by using a mixture of *β*–NaYF_4_:Yb,Tm and *β*–NaYF_4_:Yb,Er NPs. The fringes probed by the blue (Figure 1(g)), green (Figure 1(h)), and red emission (Figure 1(i)) overlap with each other with essentially the same periodicity (Figure 1(j)). In addition, the fringes are strongly dependent on the polarization direction of the incident laser; the strongest fringes can be observed under the TM mode laser excitation, while no interference fringes can be observed when a TE mode laser is used (Fig. [Supplementary-material supplementary-material-1] in the ESM). Together, these observations indicate that the fringe dispersion is not dependent on the PL emissions, but an intrinsic feature of the incident light and/or SPPs supported on Au patterns.

### 2.2. Interference Fringes between SPPs and Coherent Incident Light

**Figure 2:**
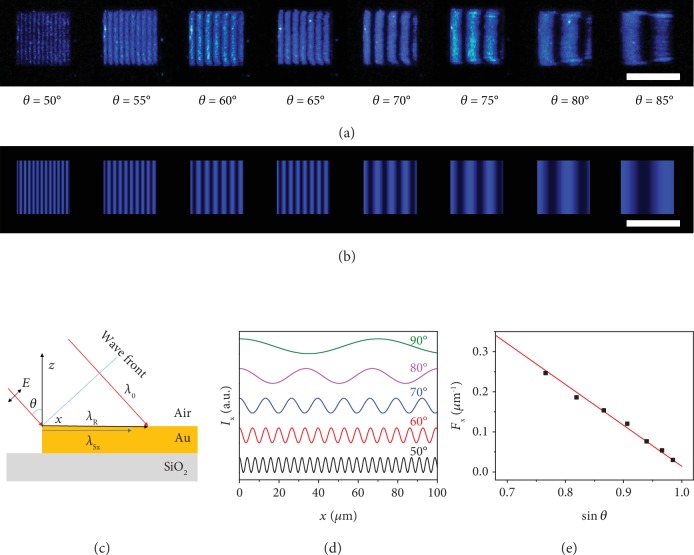
Interference fringes dispersed along the *x*-direction on Au squares with different incident angles. (a, b) Experimentally observed (a) and simulated (b) interference fringes supported by a (50 × 50) *μ*m^2^ Au square with different incident angles. (c) Schematic illustration for the excitation of SPPs on Au surface by an oblique TM laser. The incident light creates SPPs and a running wave of polarization (RWP). The interference between the SPP and the RWP forms fringes dispersed along the *x*-direction. (d) Simulated intensity dispersion of interference along the *x*-direction for different incident angles. (e) Simulated (red line) and experimentally observed (black square) spatial frequencies (*F*_*x*_) for various incident angles. The simulated fringes reproduce the experiment results precisely. Scale bar: (a, b) 50 *μ*m.

The above observations represent the first report of far-field optical visualization of such periodic fringes formed by the interference between the SPPs and the coherent incident light, which can only be observed by NSOM [24, 31] or photoemission electron microscopy [32, 33] previously. As shown in Figure 2(a), the *d*-spacing of the fringe increases considerably with increasing incident angle. In general, as illustrated in Figure 2(c), when a TM mode laser with the free-space wavelength of *λ*_0_ was obliquely shed onto the Au patterns with an incident angle of *θ* (0 < *θ* < 90°), an SPP wave could be excited at the front edge [32] and propagates along the *x*-direction with the wavelength of *λ*_S_ = *λ*_0_/*n*_eff_, where $n_{\text{eff}}=\sqrt{\left(\varepsilon_{\text{Au}}\varepsilon_{d}\right)/\left(\varepsilon_{\text{Au}}+\varepsilon_{d}\right)}$ is the effective index and *ε*_Au_ and *ε*_*d*_ are the complex dielectric constant for Au and dielectrics, respectively [1, 3]. Meanwhile, the projection of the coherent incident laser at the surface can be considered as a running wave of polarization (RWP) [24] whose amplitude varies sinusoidally along the *x*-direction with the wavelength of *λ*_R_ = *λ*_0_/sin*θ*. Note that *n*_eff_ > 1, sin*θ* < 1, and thus *λ*_S_ < *λ*_0_ < *λ*_R_. At the front edge of Au pattern (*x* = 0), both SPP and RWP waves share the same angular frequency and initial phase, which is determined by the incident light. The interference between these two coherent waves with distinct wavelengths will create a localized electric-field oscillation defined by

(1) $$\begin{array}{c} E_{x}\left(x,t\right)=E_{\text{S}x,0}e^{-x/\left(2L\right)}\sin\left(k_{\text{S}x}x+\omega_{0}t+\varphi_{0}\right)+E_{\text{R}}\sin\left(k_{\text{R}}x+\omega_{0}t+\varphi_{0}\right), \end{array}$$

where *k*_S*x*_ and *k*_R_ are the wave vectors for SPPs and RWP, *L* is the propagation length of SPPs, *E*_S*x*,0_ is the initial amplitude for SPPs at *x* = 0, *E*_R_ is the amplitude for RWP, and *φ*_0_ is the initial phase [21]. The optical near-field intensity dispersion for the interference fringes along the *x-*direction is

(2) $$\begin{array}{c} I_{x}=I_{\text{S}x,0}e^{-x/L}+2\sqrt{I_{\text{S}x,0}I_{\text{R}}}e^{-x/\left(2L\right)}\cos\left[\left(k_{\text{S}x}-k_{\text{R}}\right)x\right]+I_{\text{R}}, \end{array}$$

where *I*_S*x*,0_ = (*E*_S*x*,0_)^2^ is the initial intensity for the SPPs at the front edge (*x* = 0), and *I*_R_ = (*E*_R_)^2^ is the intensity of the RWP, which can be considered as a constant since the incident beam is evenly shed on the whole 2D pattern. Note that the intensity dispersion function is time-independent, allowing the direct observation of steady interference fringes on Au patterns (Figures 1(b)–1(j)). By substituting the definition of the wave vectors into equation (2), we can obtain

(3) $$\begin{array}{c} I_{x}=I_{\text{S}x,0}e^{-x/L}+I_{\text{R}}+2\sqrt{I_{\text{S}x,0}I_{\text{R}}}e^{-x/\left(2L\right)}\cos\left[\frac{2\pi }{\lambda_{0}}\left(n_{\text{eff}}-\sin\theta \right)x\right]. \end{array}$$

By assuming *L* → +∞ and *I*_S*x*,0_/*I*_R_ = 1 for simplification, the periodic intensity modulation along the *x*-direction can be readily simulated, with a nearly perfect match with the experimental interference fringes observed under different incident angles (Figures 2(a) and 2(b)).

According to equation (3), the spatial period (*d*-spacing) and frequency for the interference fringes along the *x*-direction are defined by

(4) $$\begin{array}{c} D_{x}=\frac{\lambda_{0}}{n_{\text{eff}}-\sin\theta }, \end{array}$$

(5) $$\begin{array}{c} F_{x}=\frac{n_{\text{eff}}-\sin\theta }{\lambda_{0}}. \end{array}$$

The *d*-spacing and frequency of the interference fringes are functions of the free-space wavelength, incident angle, and effective index, suggesting that the period of the interference fringes increases with the increasing incident angle (Figure 2(d)). The modulation frequency *F*_*x*_ and sin*θ* exhibit a linear relationship (Figure 2(e)), which is further confirmed by experimental observations, demonstrating that the proposed model can provide a sound foundation for further understanding and modulation of the SPP dispersion, propagation, and interference.

### 2.3. Attenuation of SPPs along the Propagation Direction

**Figure 3:**
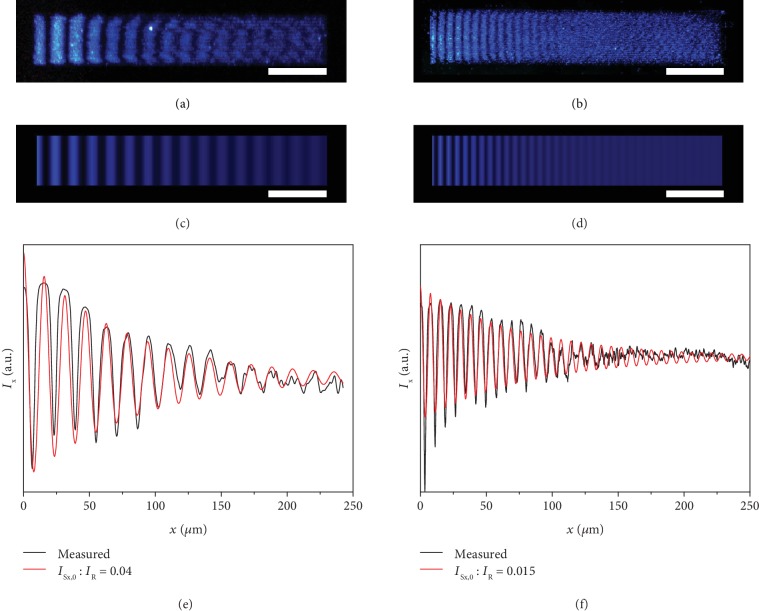
Attenuation of SPPs along the propagation direction. (a, b) PL images for the interference fringes along the *x*-direction on a Au stripe with the incident angle of (a) 70° and (b) 60°, respectively. (c, d) Simulated patterns corresponding to the interference fringes shown in (a) and (b), respectively. (e, f) Experimentally measured (black) and simulated (red) intensity dispersion for the interference fringes show in (a, c) and (b, d), respectively. *λ*_0_ = 980 nm. Scale bar: (a–d) 50 *μ*m.

In addition to periodic modulation, it is also apparent that the overall UCPL intensity attenuates along the propagating direction on a long Au stripe (Figures 3(a) and 3(b)). Plots of the intensity dispersion along the *x*-direction under the incident angles of 70 and 60° show clearly periodic modulation and gradual attenuation of the modulation amplitude (black lines in Figures 3(e) and 3(f)). To quantitatively analyze the intensity dispersions, we need to consider the attenuation effects of SPPs and the initial intensity ratio between SPPs and RWP (*I*_S*x*,0_/*I*_R_), which is determined by the excitation efficiency for SPPs. Our model provides an accurate pathway to derive the propagation length (*L*) and intensity ratio (*I*_S*x*,0_/*I*_R_) of SPPs by fitting the interference intensity dispersion with equation (3). Through numerical simulations of the observed interference patterns, we can obtain the two important SPP parameters of *L* = (50.3 ± 2.6) *μ*m and *I*_S*x*,0_/*I*_R_ = 0.04 when *θ* = 70° (Figure 3(e)) and *L* = (47.3 ± 2.5) *μ*m and *I*_S*x*,0_/*I*_R_ = 0.015 when *θ* = 60° (Figure 3(f)). Such results reveal that the propagation length values on the Au stripes are independent on the incident angle of the laser and are comparable to the theoretical and experimental results measured through NSOM previously [1, 3, 7, 20, 24]. With these fitting parameters, the 2D intensity distribution image can be regenerated (Figures 3(c) and 3(d)), which match well with the experimental UCPL images (Figures 3(a) and 3(b)). In accordance with previous experimental and theoretical results [31, 34], our observations prove that the propagation length shows no statistically significant differences at various incident angles (Fig. [Supplementary-material supplementary-material-1] in the ESM) [3]. However, the SPP excitation efficiency shows a significant polarization sensitivity [35]. At higher incident angle, the component of the incident light polarized perpendicularly to the Au surface becomes larger so that higher SPP excitation efficiency can be achieved (Fig. [Supplementary-material supplementary-material-1] in the ESM). Compared to previous methods heavily relying on the invasive and complicated NSOM protocols, our upconversion strategy proves to be a simple yet accurate far-field approach to obtain the key parameters, the attenuation length, and the excitation efficiency of SPPs.

### 2.4. Interference Fringes on Au Patterns with Various 2D Morphologies

**Figure 4:**
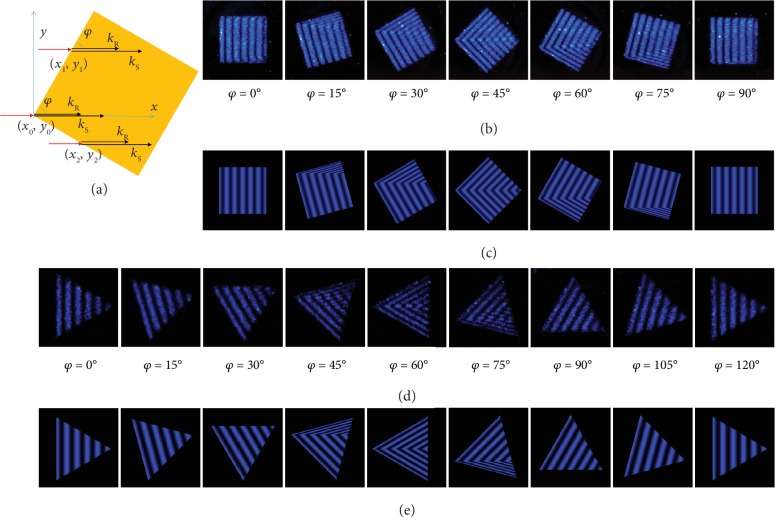
Interference fringes supported on Au patterns with various orientations. (a) Schematic illustration for the interference between SPPs and RWP on a rotated Au square with an azimuthal angle of *φ*. (b, c) Experimentally observed (b) and theoretically simulated (c) interference fringes supported on a (50 × 50) *μ*m^2^ Au square with different rotation angles. (d, e) Experimentally observed (d) and simulated (e) interference fringes on a rotated Au triangle (edge length: 50 *μ*m). *θ* = 60°.

Another important parameter for the excitation and propagation of SPPs is the azimuthal angle of the incident light. As indicated by Figure 4(a), SPPs can be excited at each point (*x*_*i*_, *y*_*i*_) on the two edges of a rotated Au square and propagate along the *x*-direction. For each horizontal line (*y* = *y*_*i*_), the interference between SPPs and RWP still follows equations (1) and (2). Therefore, the overall interference fringes on a rotated pattern can be obtained by integrating the intensity dispersion along each horizontal line, that is,

(6) $$\begin{array}{c} I_{x}\left(y=y_{i}\right)=I_{\text{S}x,0}e^{-x/L}+2\sqrt{I_{\text{S}x,0}I_{\text{R}}}e^{-x/\left(2L\right)}\cos\left[\left(k_{\text{S}x}-k_{\text{R}}\right)\left(x-x_{i}\right)\right]+I_{\text{R}}, \end{array}$$

where *x*_*i*_ is the *x*-coordinate of the starting point at the front edge for each *y* = *y*_*i*_ line. According to equation (6), we can predict that for Au patterns with any 2D morphologies, the *x*-direction interference fringes will exactly inherit the shapes of the front edges where SPPs are stimulated. As shown in Figures 4(b) and 4(c), the observed and simulated fringes on a rotated Au square fit each other well for all azimuthal angles. This principle stands for Au patterns with all other morphologies, including triangles (Figures 4(d) and 4(e)), disks (Figure 1(e)), rings (Figure 1(f)), and even irregular patterns (Fig. [Supplementary-material supplementary-material-1] in the ESM).

### 2.5. Superposition of Vertical and Lateral Interference Fringes

The Au patterns can also support a series of lateral fringes with much smaller spatial periods (Figs. [Supplementary-material supplementary-material-1]–[Supplementary-material supplementary-material-1] in the ESM). Previous theoretical simulations suggest that narrow metal stripes can support several modes of lateral SPPs [36]. Our studies show that clear lateral fringes can be observed when two parallel lateral edges of Au patterns are simultaneously illuminated by the incident beam (Fig. [Supplementary-material supplementary-material-1] in the ESM), providing a direct evidence for the strong lateral confinement effect on SPPs supported by metal stripes with finite width (Fig. [Supplementary-material supplementary-material-1] in the ESM) [36]. The total intensity dispersion for the interference fringes is the superposition of the *x*-direction interference between SPPs and RWP and the *y-*direction interferences between two SPP waves travelling in opposite directions (Fig. [Supplementary-material supplementary-material-1] in the ESM). The fitted interference fringes reproduce the observed results with high fidelity, demonstrating that UCNPs can function as effective optical probes for directly visualizing the excitation, dispersion, propagation, and interference properties of SPPs.

Together, UCNPs can upconvert the invisible, evanescently confined SPP fields into visible PL images, allowing far-field visualizing the interference fringes formed by SPPs and the coherent incident light. UCNPs represent a novel kind of PL probes to reveal the intrinsic dependence of SPP excitation and propagation on several basic factors (*λ*_0_, *θ*, *φ*, *ε*_*m*_, and *ε*_*d*_) in complicated environments. Our method provides a facile far-field approach for determining the key SPP parameters (excitation efficiency, propagation length), which can play a critical and indispensable role in SPP-related research and practices.

### 2.6. Interference Fringes as Ultrasensitive Spectrometer-Free Optical Ruler

Besides the significance in fundamental SPP research, the interference fringes can be also explored as an optical sensor to monitor the variations in local dielectric environment, according to equation (4). The interference fringes show a rapid and reversible response to local dielectric change that can be monitored in real-time ([Supplementary-material supplementary-material-1] in the ESM). The linear relationship between the frequency of the fringes and the effective index (equation (5)) is confirmed by experiment results over large effective index variations (Fig. [Supplementary-material supplementary-material-1] and [Supplementary-material supplementary-material-1] in the ESM). In principle, the lowest detectable refractive index variation can be defined by dividing the resolution of the far-field microscope by the refractive index sensitivity of the interference fringes. On the one hand, the *d*-spacing and the refractive index sensitivity for the interference fringes can be increased dramatically by increasing the incident angle (Fig. [Supplementary-material supplementary-material-1] in the ESM) and incident wavelength (Fig. S12 in the ESM). In particular, equation (4) reveals that the refractive index sensitivity can be enhanced significantly when (*n*_eff_ − sin*θ*) → 0. The refractive index sensitivity can be over 3000 *μ*m per refractive index unit (RIU) in air with the incident angle of 85° (Fig. [Supplementary-material supplementary-material-1] in the ESM). On the other hand, the resolution of the far-field optical microscope can also be enhanced by using short wavelength emissions as optical signal enabled by the UCPL (e.g., <500 nm). According to the Abbe diffraction limit [37], a 100x objective with the numerical aperture of 0.80 has the spatial resolution of about 300 nm for the working wavelength of 476 nm (blue emission of *β*–NaYF_4_:Yb,Tm NPs, Fig. [Supplementary-material supplementary-material-1] in the ESM). Therefore, a variation of 10^−4^ RIU can be detected when a single period of the interference fringes is measured. And a variation of 10^−5^ RIU (or even lower) can be monitored when ten or more periodically dispersed fringes were measured to obtain the average spatial period value. That is, the refractive index sensitivity of our SPP-UCPL sensors is comparable to that of complex plasmonic metamaterial sensors working in the NIR regime [11, 38]. Moreover, as compared to previous plasmonic sensors that measure the wavelength shift in response to refractive index variation in the NIR regime [11, 12, 38, 39], our approach can directly transduce the local dielectric environment changes into the variations in interference fringe periodicity, thus enabling an entirely new design of ultrasensitive spectrometer-free optical ruler for direct detection of various molecular binding events in real time.

**Figure 5:**
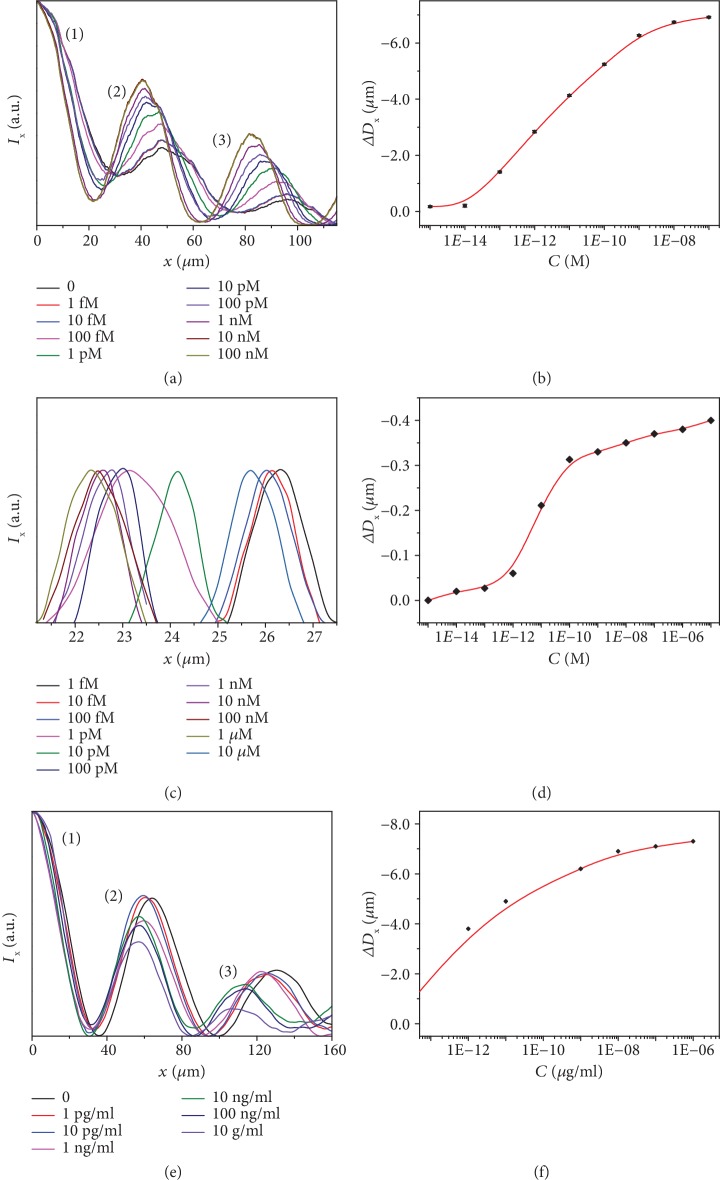
Response to streptavidin and prostate-specific antigen (PSA) of different concentrations for specifically modified Au-UCNP sensors. (a) Normalized intensity distribution of the interference fringes acquired in air after immersing the biotinylated Au-UCNP pattern in streptavidin solution of different concentrations. (b) Variation of Δ*D*_*x*_ (in air) versus streptavidin concentrations for the biotinylated Au-UCNP pattern in panel (a). (c) Normalized intensity distribution of the 10^th^ interference fringe acquired in situ when the biotinylated Au-UCNP pattern is immersed in streptavidin solution of different concentrations. (d) Variation of Δ*D*_*x*_ (in solution) versus streptavidin concentrations for the biotinylated Au-UCNP pattern in panel (c). (e) Normalized intensity distribution of the interference fringes dispersed along the *x*-direction acquired in N_2_ after the antibody-modified Au-UCNP sensor is exposed to PSA solution of different concentrations. (f) Variation of Δ*D*_*x*_ versus PSA concentrations for the antibody-modified Au pattern.

A typical biotin-streptavidin affinity model [11, 38, 39] was first employed to evaluate the sensitivity and limit of detection (LOD) of this unique spectrometer-free optical ruler. In principle, the specific binding of streptavidin to surface biotin groups can induce an increase in a local effective index, which can, in turn, lead to a decrease in the interference fringe *d*-spacing. Figures 5(a) and 5(b) and Fig. [Supplementary-material supplementary-material-1] in the ESM show the variation of the intensity dispersion for the interference fringes acquired in air after the biotinylated sensor was exposed to streptavidin solutions of different concentrations through a microfluidic configuration (Fig. [Supplementary-material supplementary-material-1] in the ESM). The peaks for intensity dispersion curves show a significant left-shift with increasing streptavidin concentration (Figure 5(a)). The variation of *d*-spacing versus the streptavidin concentration (Figure 5(b)) indicates that the LOD for streptavidin can be as low as 10 fM for the single-pattern SPP-UCPL sensor. Additionally, our direct measurement in solution reveal that streptavidin of the concentration at the pM level can be detected by the SPP-UCPL optical ruler in real-time (Figures 5(c) and 5(d) and Fig. [Supplementary-material supplementary-material-1] in the ESM). To our knowledge, previously reported LOD values to streptavidin in a wavelength-shift type of plasmonic sensors typically range from pM to *μ*M concentrations [40], requiring the help of spectrometers or other scanning components [41]. Therefore, the SPP interference fringes represent a new design of ultrasensitive, directly measurable optical rulers for biomolecule detection.

To demonstrate its broad applicability and capability, we have further employed the SPP-UCPL optical ruler for highly sensitive detection of another important biomarker, prostate-specific antigen (PSA) [42]. Most of the current PSA biosensors are derived from variations of enzyme-linked immunosorbent assays (ELISA) [43–45], requiring various exogenous label molecules for the detection, which inevitably impedes their applications in both fundamental research and healthcare diagnostics. Other studies usually require high-cost instrument [46, 47], complicated processing protocols such as DNA scanometric detection [48], and/or complex structures of sandwich immunoassay [49, 50] to reach a fM level PSA sensitivity. In contrast, with our most simplified SPP-UCPL optical ruler, a LOD of less than 1 pg/ml (30 fM) for PSA can be achieved with a simple optical observation and space measurement (Figures 5(e) and 5(f) and Fig. [Supplementary-material supplementary-material-1] in the ESM), which is comparable to previous reports that require more complicated instrumentation ([Supplementary-material supplementary-material-1] in the ESM). Together, our method based upon SPP-RWP interference fringes represents a cost-effective, directly measurable optical ruler for ultrasensitive, spectrometer-free detection and screening of specific chemical or biochemical species.

## 3. Conclusion

In summary, we have demonstrated the first utilization of RE-doped UCNPs as nanoscale optical probes that can quantitatively upconvert evanescently confined SPPs into visible emissions for real-time mapping SPPs in complicated environments with high throughput and high fidelity. The interference fringes can serve as an ultrasensitive, directly measurable optical ruler for rapid detection of local dielectric variations. A variation of the refractive index < 10^–5^ RIU can be detected directly without any bulk spectrometers or scanning components. In practice, the SPP-UCPL sensor shows a LOD about 10 fM for streptavidin and <30 fM for PSA, respectively. The integration of SPP and UCPL materials opens a new possibility to quantitatively map the SPP dispersion in complicated environments and to design a new generation of ultrasensitive, spectrometer-free sensors. We believe that our findings shall shed light on fundamental studies and applications of SPP and UCPL materials.

## Supplementary Materials

Supplementary 1 Fig. S1: TEM images and upconversion emission spectra for *β*–NaYF4:Yb,Tm and *β*–NaYF4:Yb,Er NPs. (a) TEM image and (b) upconversion emission spectra for *β*–NaYF4:Yb,Tm NPs. (c) TEM image and (d) upconversion emission spectra for *β*–NaYF4:Yb,Er NPs. The average particle size is about 55 nm for the *β*–NaYF4:Yb,Tm NPs and 60 nm for the *β*–NaYF4:Yb,Er NPs, respectively. When excited by a 980 nm continues wave diode laser, the *β*–NaYF4:Yb,Tm NPs have a blue/red emission, while the *β*–NaYF4:Yb,Er NPs give a green/red emission. The corresponding f–f transitions for each emission are identified in panels (b) and (d). Scale bar: (a) 200 nm, (c) 100 nm. Fig. S2: AFM profiles for the Au patterns and Au-UCNP composites. (a) AFM profile of bare Au surface, showing a surface roughness of 0.8 nm. (b) Large area AFM profile showing the edge of the Au-UCNP pattern. Note that there is a single layer of UCNPs close to the edge of the Au pattern. The size of the UCNP is about 55 nm, which is consistent with the TEM observations, and the height of the Au pattern is about 75 nm. (c) AFM image of UCNPs supported on the Au pattern, showing a randomly dispersed NP single-layer with a surface coverage of about 30%. (d) Enlarged AFM image of the UCNPs on Au surface, showing the size and shape uniformity for the UCNPs. Scale bar: (a, c) 1 *μ*m, (b) 2 *μ*m, and (d) 100 nm. Fig. S3: schematic illustration of the set-up for the observation of fringes formed by the interference between the incident light and the SPPs. The Au-UCNP pattern supported on a SiO_2_/Si substrate is fixed on the specimen holder of the optical microscope. A 980 nm continuous wave diode laser is obliquely shed onto the sample, and the upconversion emission is collected by the objective (10x, NA = 0.25; 50x, NA = 0.50; and 100x, NA = 0.8 LMPLFLN-BD objectives). The polarization direction of the incident beam is controlled by a polarizer and a half-wave plate. Fig. S4: interference fringes observed with different polarization directions of the incident laser. No interference patterns can be observed when the laser is in the TE mode, while the strongest interference fringes are generated by the TM mode incident light. The intensity of the incident light and exposure parameters were fixed during the whole test. Note that the intensity of the blue spot at the left-up corner in each panel, which is located on the SiO_2_/Si substrate far away from the Au pattern, remains constant and can be used as a reference for the intensity change of the interference fringes. Scale bar: 25 *μ*m. Fig. S5: interference intensity dispersion observed at different incident angles. (a) Experimentally measured intensity dispersion of interference fringes along the *x*-direction on a Au stripe at different incident angles. (b) The fitted SPP propagation length versus the incident angle curve, showing no statistically significant variation among the propagation length values at different incident angles. (c) Relative intensity ratios (fitted) between the initial SPP and the RWP (*I*_S*x*,0_ : *I*_R_) or the incident light (*I*_S*x*,0_ : *I*_0_), showing an increase of SPP excitation efficiency with the increase of the incident angle. The intensity of the incident light is calculated by *I*_R_ = *I*_0_cos*θ*. Error bars represent the standard deviations from three independent experiments. Fig. S6: interference fringes supported by an irregular Au pattern. (a) Bright-field image of the irregular Au pattern. (b) PL image for the interference fringes supported on the Au pattern. The shape of the fringes follows the front (right) edges of the Au pattern. Scale bar: 50 *μ*m. Fig. S7: interference fringes supported on different Au patterns showing the edge effects on the fringes. (a) Au patterns with several bevel edges, (b, c) L-shape Au pattern, and (d) rectangle Au pattern. The red dashed line highlights the boundary between different fringes. In panels (a)–(c), no lateral fringes can be observed since there are no, or only one, lateral edge that is excited by the incident light. However, in panel (d), clear lateral fringes are observed because both lateral edges are excited by the incident light to create two SPPs travelling along the opposite direction to form the interference fringes. Scale bar: (a, b) 50 *μ*m, (c, d) 20 *μ*m. Fig. S8: interference fringes supported by Au stripes with different widths. (a, b) PL images showing the lateral interference fringes on Au stripes with the width of 5 *μ*m (a) and 50 *μ*m (b), respectively. (c) PL image showing both the vertical and lateral interference fringes on Au stripes with different widths. (d) The *d*-spacing for the lateral fringes versus the width curve for lateral fringes supported on Au stripes with different widths, showing the lateral confinement effects when the Au stripe width is smaller than 20 *μ*m. Scale bar: (a, b) 10 *μ*m, (c) 50 *μ*m. Fig. S9: vertical and lateral interference fringes supported on Au stripes. (a, b) Observed (a) and simulated fringes (b) supported on a 50 *μ*m wide Au stripe. (c, d) Corresponding intensity dispersion along the *x*- and *y*-directions for observed and simulated fringes shown in (a, b). The measured (black) and simulated (red) curves fit each other precisely. *θ* = 60°. Scale bar: (a, b) 20 *μ*m. Fig. S10: response to different solvents. (a) Interference fringe (*x*-direction) frequency versus effective index (neff) curve for different surrounding media (air, methanol, water, ethanol, isopropanol, 1-butanol, and DMF). (b) PL images for the interference fringes supported by a 50 *μ*m wide Au stripe immersed in different dielectrics. The period for the fringes decreases with increasing the effective index by changing to solvents with higher dielectric constant and recovers after the solvent is evaporated by N2 flow. The free-space wavelength is 980 nm, and the incident angle is 60°. Fig. S11: calculated *D*_*x*_–*n*_d_ relationship at different incident angles. (a–c) *D*_*x*_–*n*_d_ curves at different incident angles. (d–f) The absolute value of the slope for the *D*_*x*_–*n*_d_ curve (i.e., refractive index sensitivity) is plotted for different incident angles, showing that the refractive index sensitivity increases dramatically with the increase of the incident angle. Fig. S12: influence of the incident light wavelength on the periods of the interference fringes. (a) Calculated *d*-spacing (period) values of the *x*-direction interference fringes (if present) versus the free-space wavelength curves for different incident angles, showing that the *d*-spacing will increase at all incident angles when the incident free-space wavelength is increased. (b, c) Interference fringes supported by a 50 *μ*m wide Au stripe excited by a (b) 808 nm or (c) 980 nm laser with the same incident angle (~75°), confirming the calculation results shown in panel (a). Another type of UCNPs, *β*–NaGdF4:Yb,Er@NaGdF4:Nd NPs, which can be excited by either 808 or 980 nm laser1, were used to probe the intensity dispersion of the interference fringes under incident light with the free-space wavelength of 808 or 980 nm, respectively. Scale bar: 25 *μ*m. Fig. S13: real-color image of the interference fringes showing the response to streptavidin for biotinylated Au-UCNP sensors. The fringes supported on the biotinylated Au-UCNP pattern show a significant systematic shift (decrease of *d*-spacing) after the binding of streptavidin by surface biotin groups. For the sensing of streptavidin at each bulk concentration, after the binding of surface biotin and streptavidin in the microfluidic channel (illustrated in Fig. S14), the Au-UCNP pattern and the channel are rinsed with DI water for three times to remove excessive unspecifically bound streptavidin molecules and dried under N2 flow. PL images were collected to verify that stable fringes were obtained for the calculation of spatial periods. The three bright stripes highlighted correspond to the three intensity peaks shown in Figure 5(a) in the main text. Scale bar: 50 *μ*m. Fig. S14: schematic illustration for the microfluidic channels used in the solvent and streptavidin sensing experiments. The biotinylated Au-UCNP pattern supported on a SiO_2_/Si substrate is placed in a PDMS channel with a volume of about 10 *μ*l, which is sealed with a cover slide (0.15 mm in thickness) and tightened with clamps. The sample is fixed on the specimen holder of the optical microscope. A 980 nm laser is obliquely shed onto the sample and the upconversion emission is collected by the objective. Different solvents or solutions with different streptavidin concentrations were injected by syringe pumps. N2 flow was used to blow dry the samples before changing different solvents. Fig. S15: response to streptavidin solution of different concentrations for biotinylated Au-UCNP sensors. Directly observed interference fringes supported on the Au-UCNP square immersed in air or aqueous solution of streptavidin with different concentrations. Scale bar: 20 *μ*m. Fig. S16: real-color image of the interference fringes showing the response to PSA for biotinylated Au-UCNP sensors. The fringes supported on the antibody-modified Au-UCNP pattern show a significant systematic shift (decrease of *d*-spacing) after the binding of PSA by surface antibody groups. For the sensing of PSA at each bulk concentration, after the binding of surface antibody and PSA in the microfluidic channel (illustrated in Fig. S13), the Au-UCNP pattern and the channel are rinsed with DI water for three times to remove excessive nonspecifically bound PSA molecules and dried under N2 flow. PL images were collected to verify that stable fringes were obtained for the calculation of spatial periods. The three bright stripes highlighted correspond to the three intensity peaks shown in Figure 5(e) in the main text. Scale bar: 100 *μ*m. Fig. S17: time-dependent intensity dispersion for the interference between the RWP and the SPPs in one period, showing the envelope curve (low frequency, cyan) and the time-dependent intensity curves (high frequency) for 2*ω*0*t* = 0 (black), *π*/2 (red), *π* (green), and 3*π*/2 (blue), respectively. The incident light beam (*λ*0 = 980 nm) is obliquely shed onto the surface at *θ* = 60°. Fig. S18: conversion from UCPL intensity to local field intensity. (a) Emission intensity versus excitation power density curves for the 476 nm emission of *β*–NaYF4:Yb,Tm NPs when excited by a 980 nm CW laser. The slope for the Ln(*I*_476_)–Ln(*P*) curve is 3.03. (b) Measured UCPL intensity (476 nm) dispersion, local field intensity converted based on the $I_{\text{LF}}\propto \sqrt[{3}]{I_{476}}$ relationship, and simulated intensity dispersion curves. Table S1: calculated period of the interference fringe patterns in different solvents. Table S2: calculated period of the interference fringe patterns on air/Au surface with different incident wavelengths and angles. Table S3: comparison of different PSA detection methods.

Supplementary 2 Real-time observation of the interference fringes in response to the local dielectric change. The surrounding environment for the Au-UCNP stripe was changed from air to butanol and then to air again during the observation.
