# Supplementary material for: Quantitative Surface Plasmon Interferometry via Upconversion Photoluminescence Mapping
Source: Research (Wash D C). 2019 Sep 15;2019:8304824. doi: 10.34133/2019/8304824 (PMC6946267; doi:10.34133/2019/8304824)
Supplement: Supplementary 1 — Fig. S1: TEM images and upconversion emission spectra for β–NaYF4:Yb,Tm and β–NaYF4:Yb,Er NPs. (a) TEM image and (b) upconversion emission spectra for β–NaYF4:Yb,Tm NPs. (c) TEM image and (d) upconversion emission spectra for β–NaYF4:Yb,Er NPs. The average particle size is about 55 nm for the β–NaYF4:Yb,Tm NPs and 60 nm for the β–NaYF4:Yb,Er NPs, respectively. When excited by a 980 nm continues wave diode laser, the β–NaYF4:Yb,Tm NPs have a blue/red emission, while the β–NaYF4:Yb,Er NPs give a green/red emission. The corresponding f–f transitions for each emission are identified in panels (b) and (d). Scale bar: (a) 200 nm, (c) 100 nm. Fig. S2: AFM profiles for the Au patterns and Au-UCNP composites. (a) AFM profile of bare Au surface, showing a surface roughness of 0.8 nm. (b) Large area AFM profile showing the edge of the Au-UCNP pattern. Note that there is a single layer of UCNPs close to the edge of the Au pattern. The size of the UCNP is about 55 nm, which is consistent with the TEM observations, and the height of the Au pattern is about 75 nm. (c) AFM image of UCNPs supported on the Au pattern, showing a randomly dispersed NP single-layer with a surface coverage of about 30%. (d) Enlarged AFM image of the UCNPs on Au surface, showing the size and shape uniformity for the UCNPs. Scale bar: (a, c) 1 μm, (b) 2 μm, and (d) 100 nm. Fig. S3: schematic illustration of the set-up for the observation of fringes formed by the interference between the incident light and the SPPs. The Au-UCNP pattern supported on a SiO2/Si substrate is fixed on the specimen holder of the optical microscope. A 980 nm continuous wave diode laser is obliquely shed onto the sample, and the upconversion emission is collected by the objective (10x, NA = 0.25; 50x, NA = 0.50; and 100x, NA = 0.8 LMPLFLN-BD objectives). The polarization direction of the incident beam is controlled by a polarizer and a half-wave plate. Fig. S4: interference fringes observed with different polarization directions of [file 8304824.f1.docx]

Electronic Supplementary Material for

**Quantitative Surface Plasmon Interferometry via Upconversion Photoluminescence mapping**

*Anxiang Yin,^1,6†^ Hao Jing,^1†^ Zhan Wu,^2,3^ Qiyuan He,^1^ Yiliu Wang,^1^ Zhaoyang Lin,^1^ Yuan Liu,^2^ Mengning Ding,^2^ Xu Xu,^1^ Zhe Fei^5^, Jianhui Jiang,^3^ Yu Huang,^2,4^ Xiangfeng Duan^1,4^**

*^1^ Department of Chemistry and Biochemistry, University of California, Los Angeles, California 90095, USA.*

*^2^ Department of Materials Science and Engineering, University of California, Los Angeles, California 90095, USA.*

*^3^ State Key Laboratory for Chemo/Biosensing and Chemometrics, College of Chemistry and Chemical Engineering, Hunan University, Changsha 410082, China.*

*^4^ California NanoSystems Institute, University of California, Los Angeles, California 90095, USA.*

*^5^ Department of Physics & Astronomy, Iowa State University, Ames, Iowa 50011, USA*

*^6^ School of Chemistry and Chemical Engineering, Beijing Institute of Technology, Beijing 100008, China*

**Correspondence to: xduan@chem.ucla.edu (X.D.)*

*† These authors contributed equally to this work.*

**Supplementary Data**

**
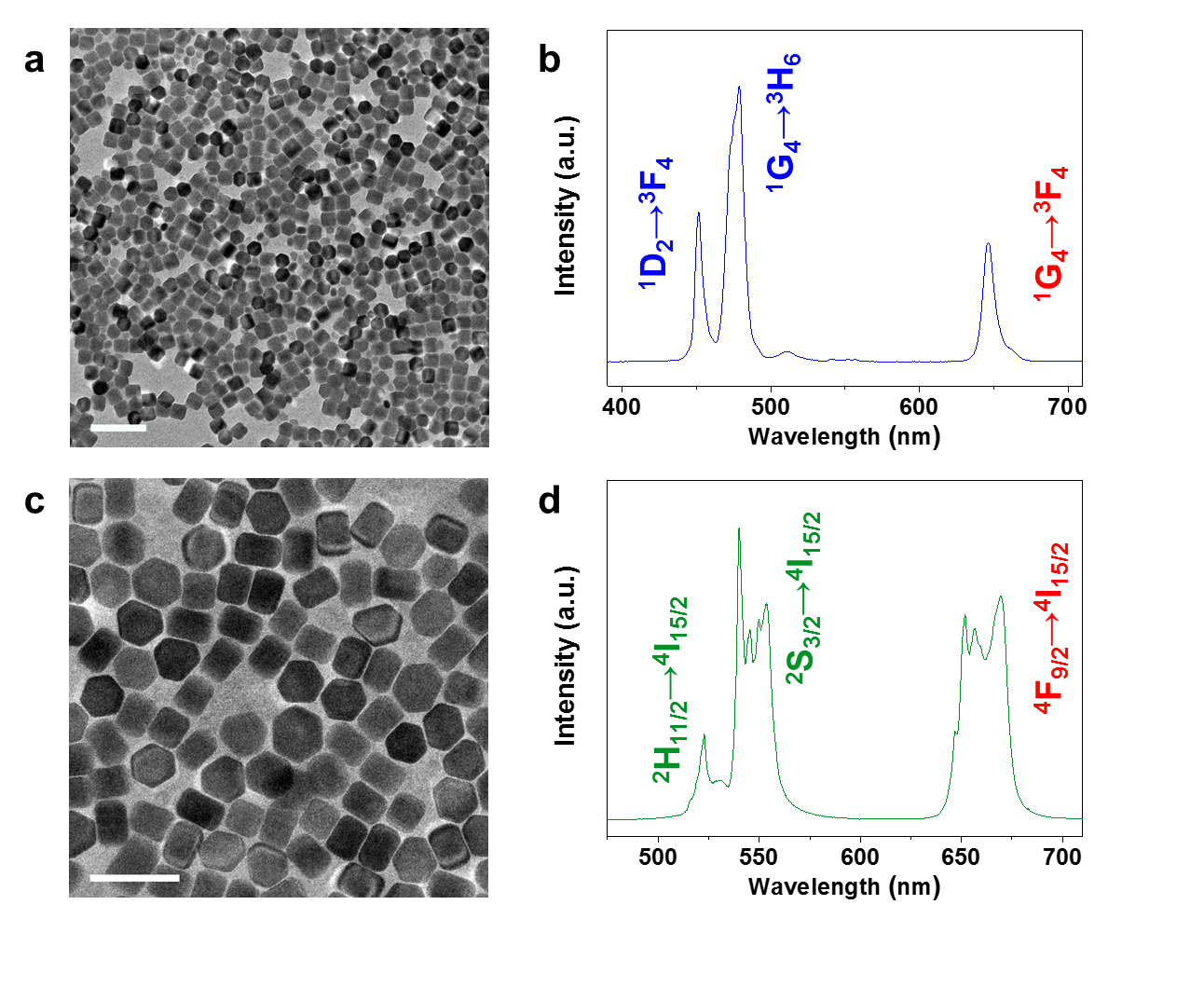
**

**Fig. S1.** **TEM images and upconversion emission spectra for *β*–NaYF_4_:Yb,Tm and *β*–NaYF_4_:Yb,Er NPs.** (a) TEM image and (b) upconversion emission spectra for *β*–NaYF_4_:Yb,Tm NPs. (c) TEM image and (d) upconversion emission spectra for *β*–NaYF_4_:Yb,Er NPs. The average particle size is about 55 nm for the *β*–NaYF_4_:Yb,Tm NPs, and 60 nm for the *β*–NaYF_4_:Yb,Er NPs, respectively. When excited by a 980 nm continues wave diode laser, the *β*–NaYF_4_:Yb,Tm NPs have a blue/red emission, while the *β*–NaYF_4_:Yb,Er NPs give a green/red emission. The corresponding *f*–*f* transitions for each emission are identified in panels (b) and (d). Scale bar: (a) 200 nm, (c) 100 nm.


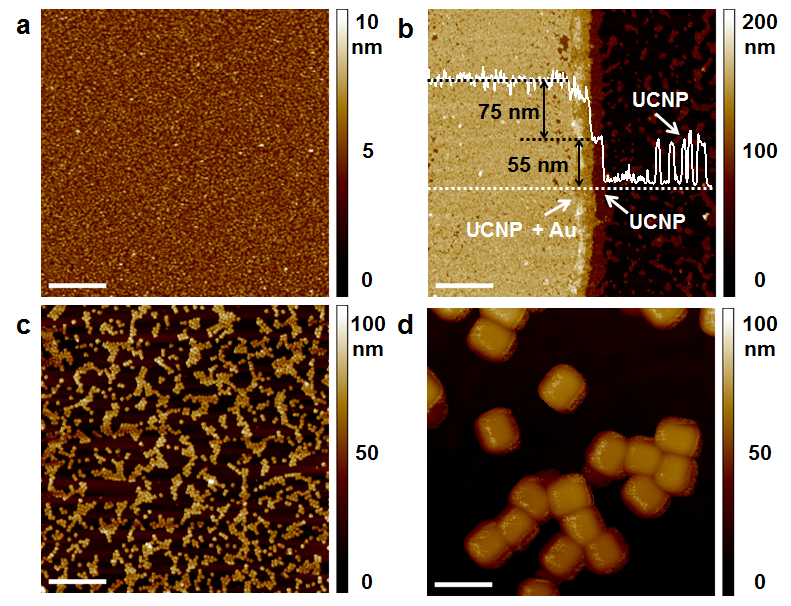


**Fig. S2.** **AFM profiles for the Au patterns and Au–UCNP composites.** (a) AFM profile of bare Au surface, showing a surface roughness of 0.8 nm. (b) Large area AFM profile showing the edge of the Au–UCNP pattern. Note that there is a single layer of UCNPs close to the edge of the Au pattern. The size of the UCNP is about 55 nm, which is consistent with the TEM observations, and the height of the Au pattern is about 75 nm. (c) AFM image of UCNPs supported on the Au pattern, showing a randomly dispersed NP single-layer with a surface coverage of about 30%. (d) Enlarged AFM image of the UCNPs on Au surface, showing the size and shape uniformity for the UCNPs. Scale bar: (a,c) 1 µm, (b) 2 µm, and (d) 100 nm.


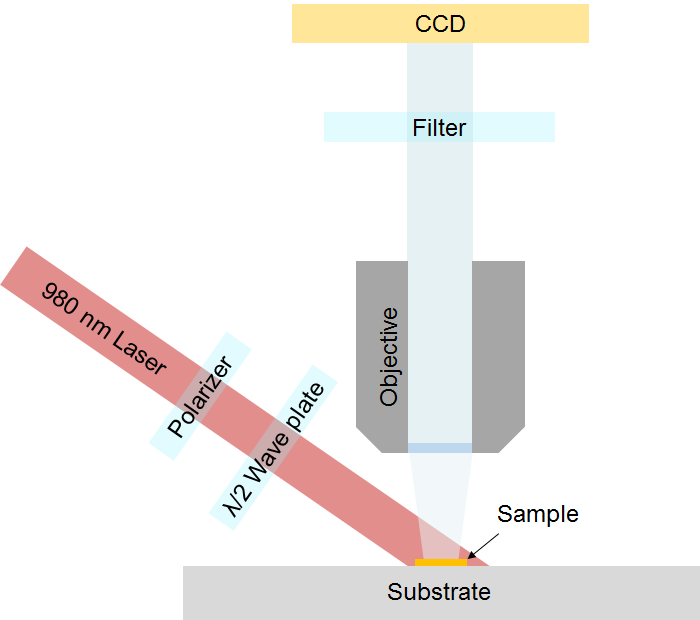


**Fig. S3. Schematic illustration of the set-up for the observation of fringes formed by the interference between the incident light and SPPs.** The Au–UCNP pattern supported on a SiO_2_/Si substrate is fixed on the specimen holder of the optical microscope. A 980 nm continuous wave diode laser is obliquely shed onto the sample and the upconversion emission is collected by the objective (10 ×, NA = 0.25; 50 ×, NA = 0.50; and 100 ×, NA = 0.8 LMPLFLN-BD objectives). The polarization direction of the incident beam is controlled by a polarizer and a half-wave plate.


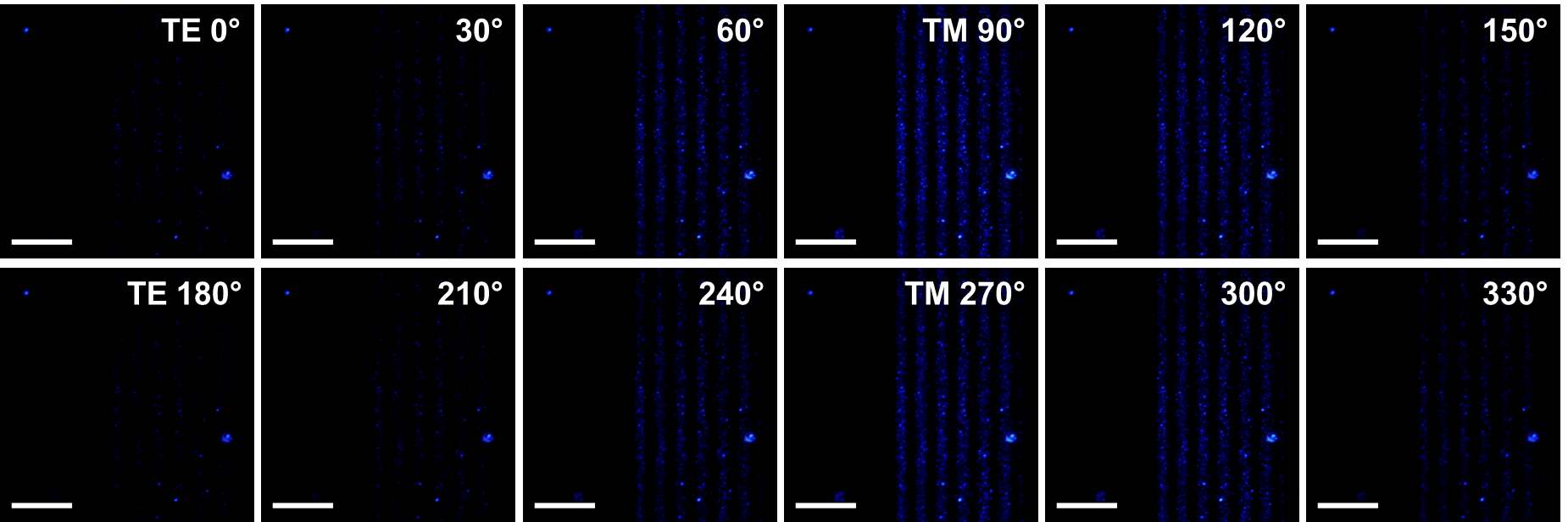


**Fig. S4. Interference fringes observed with different polarization directions of the incident laser.** No interference patterns can be observed when the laser is in TE mode, while strongest interference fringes are generated by the TM mode incident light. The intensity of the incident light and exposure parameters were fixed during the whole test. Note that the intensity of the blue spot at the left-up corner in each panel, which is located on the SiO_2_/Si substrate far away from the Au pattern, remains constant and can be used as a reference for the intensity change of the interference fringes. Scale bar: 25 µm.


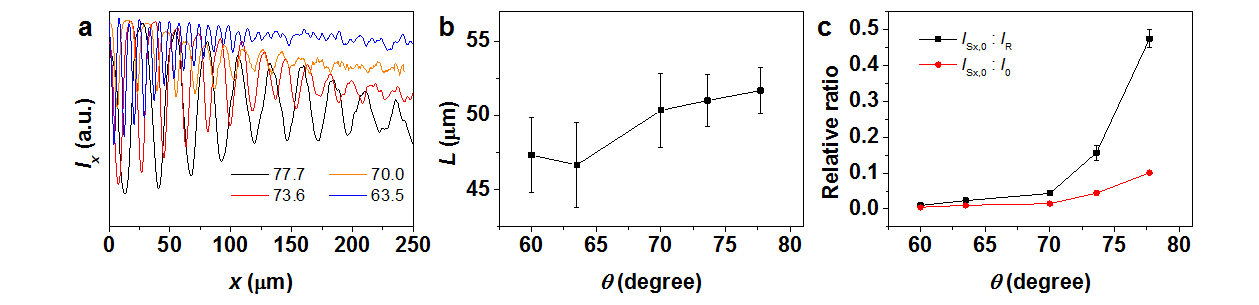


**Fig. S5. Interference intensity dispersion observed at different incident angles.** (a) Experimentally measured intensity dispersion of interference fringes along the *x*-direction on a Au stripe at different incident angles. (b) The fitted SPP propagation length versus incident angle curve, showing no statistically significant variation among the propagation length values at different incident angles. (c) Relative intensity ratios (fitted) between the initial SPP and the RWP (*I*_Sx,0_ : *I*_R_) or the incident light (*I*_Sx,0_ : *I*_0_), showing an increase of SPP excitation efficiency with the increase of the incident angle. The intensity of the incident light is calculated by *I*_R_ = *I*_0_cos*θ*. Error bars represent the standard deviations from three independent experiments.


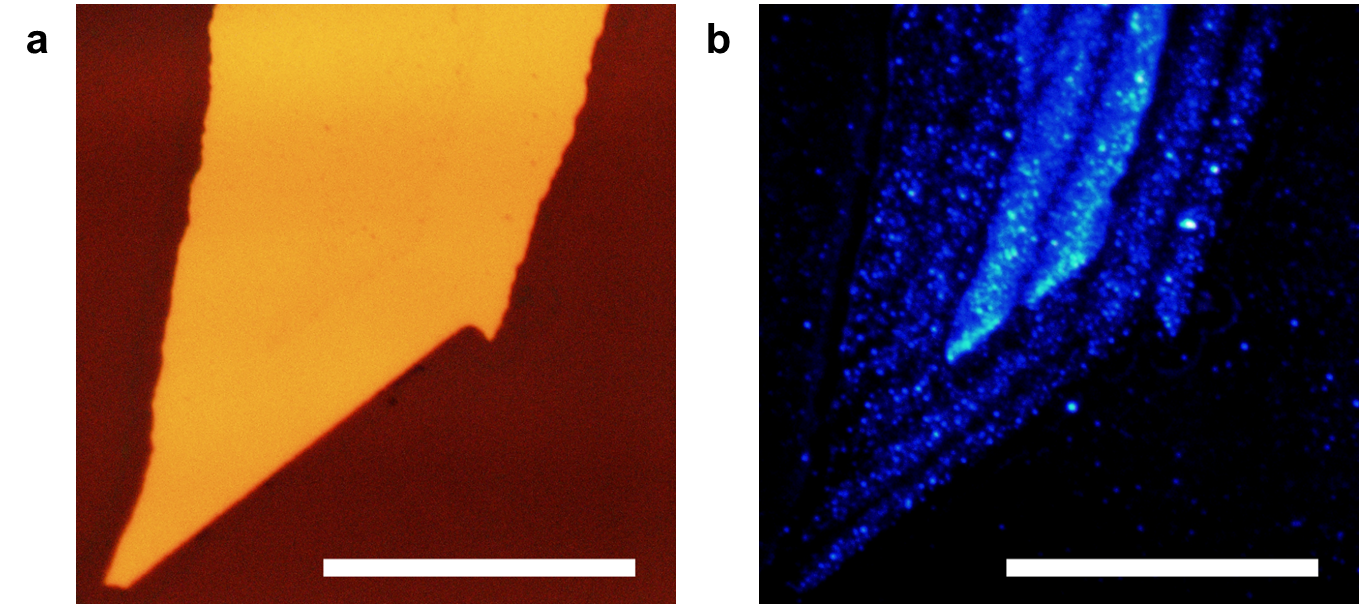


**Fig. S6. Interference fringes supported by an irregular Au pattern.** (a) Bright-field image of the irregular Au pattern. (b) PL image for the interference fringes supported on the Au pattern. The shape of the fringes follows the front (right) edges of the Au pattern. Scale bar: 50 µm.


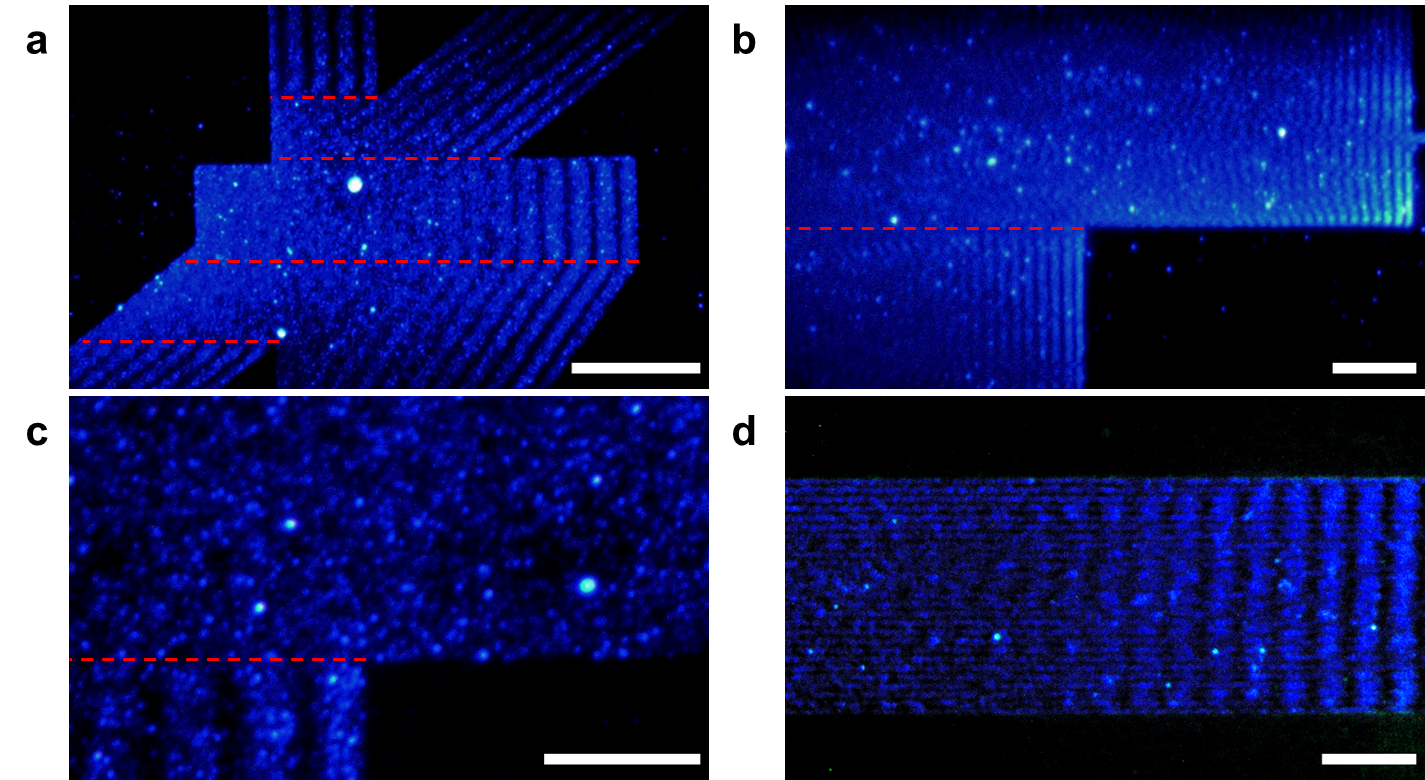


**Fig. S7. Interference fringes supported on different Au patterns showing the edge effects on the fringes.** (a) Au patterns with several bevel edges, (b,c) L-shape Au pattern and, (d) rectangle Au pattern. The red dashed line highlights the boundary between different fringes. In panels a–c, no lateral fringes can be observed since there are no, or only one, lateral edge that is excited by the incident light. However, in panel d, clear lateral fringes are observed because both lateral edges are excited by the incident light to create two SPPs travelling along the opposite direction to form the interference fringes. Scale bar: (a,b) 50 µm, (c,d) 20 µm.


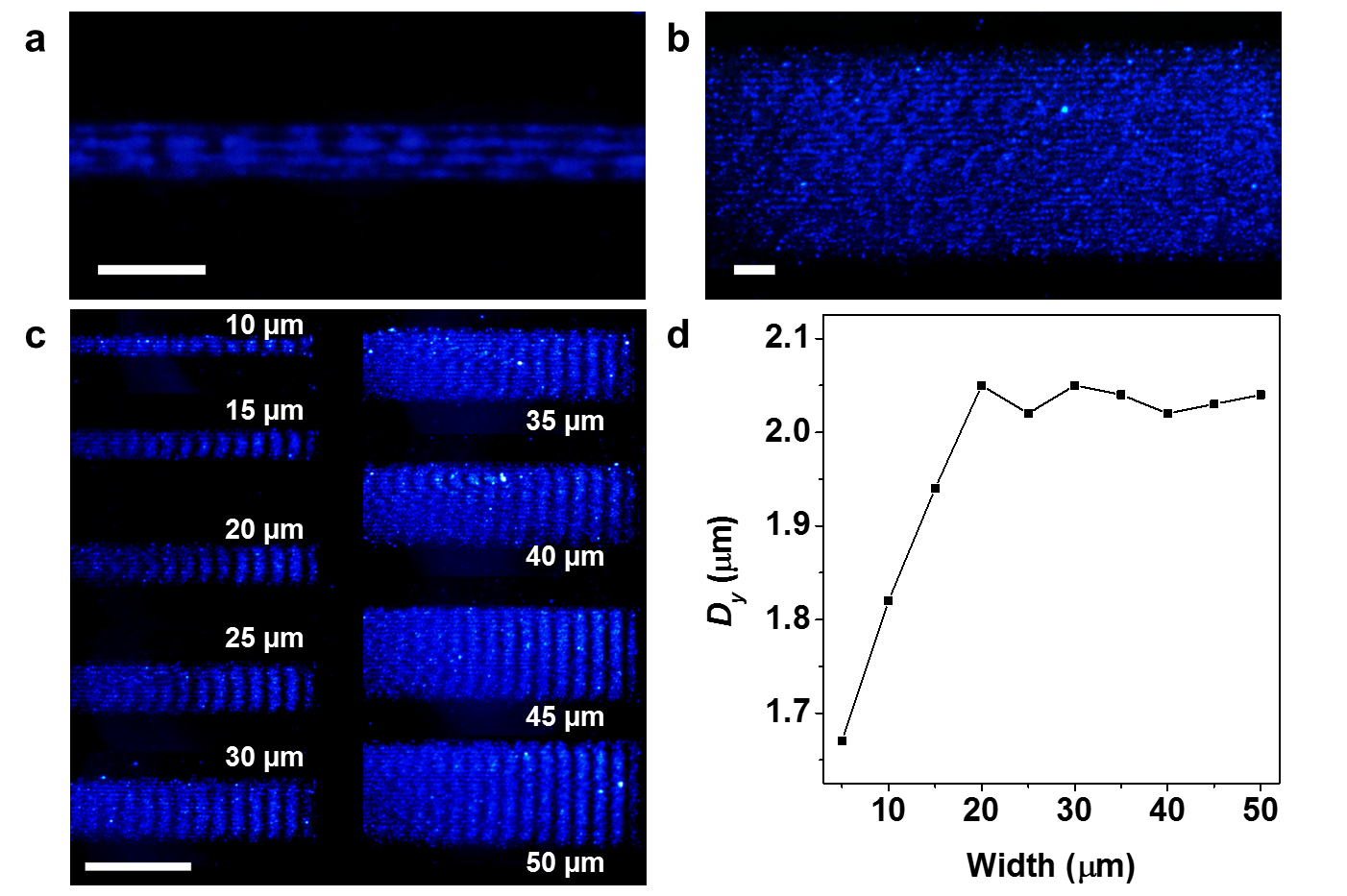


**Fig. S8. Interference fringes supported by Au stripes with different widths.** (a,b) PL images showing the lateral interference fringes on Au stripes with the width of 5 µm (a) and 50 µm (b), respectively. (c) PL image showing both the vertical and lateral interference fringes on Au stripes with different widths. (d) The *d*-spacing for the lateral fringes versus width curve for lateral fringes supported on Au stripes with different widths, showing the lateral confinement effects when the Au stripe width is smaller than 20 µm. Scale bar: (a,b) 10 µm, (c) 50 µm.


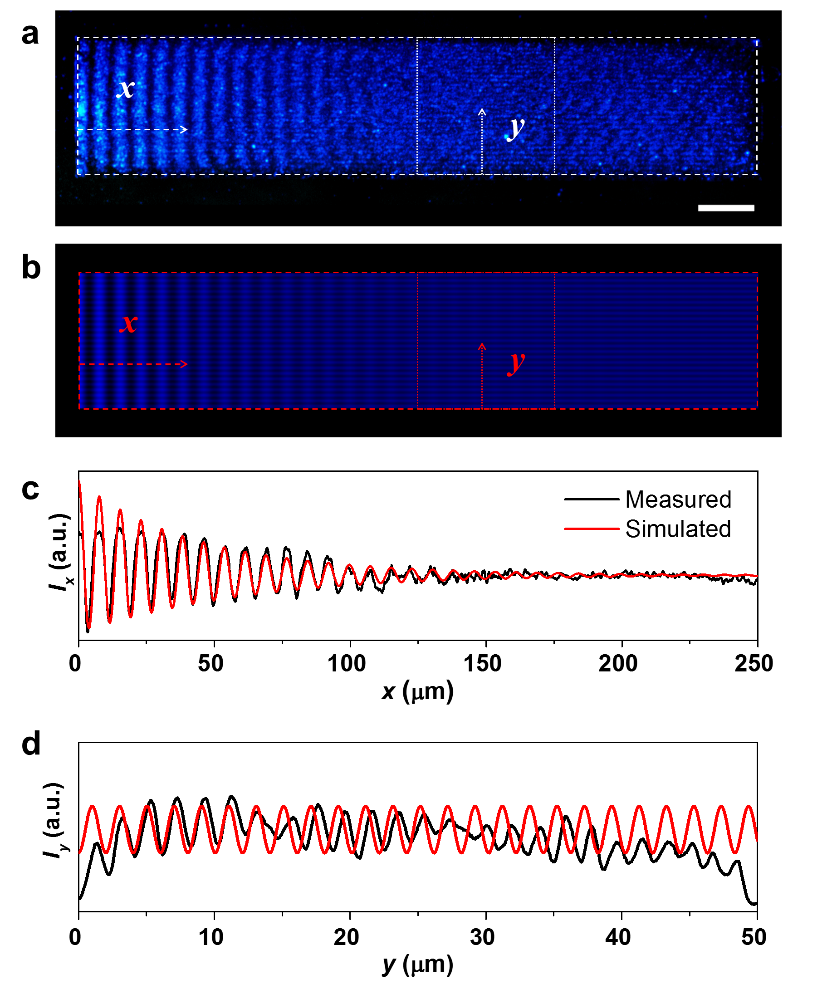


**Fig. S9.** **Vertical and lateral interference fringes supported on Au stripes.** (a,b) Observed (a) and simulated fringes (b) supported on a 50-µm-wide Au stripe. (c,d) Corresponding intensity dispersion along the *x*- and *y*-directions for observed and simulated fringes shown in (a,b). The measured (black) and simulated (red) curves fit each other precisely. *θ* = 60°. Scale bar: (a,b) 20 µm.

**
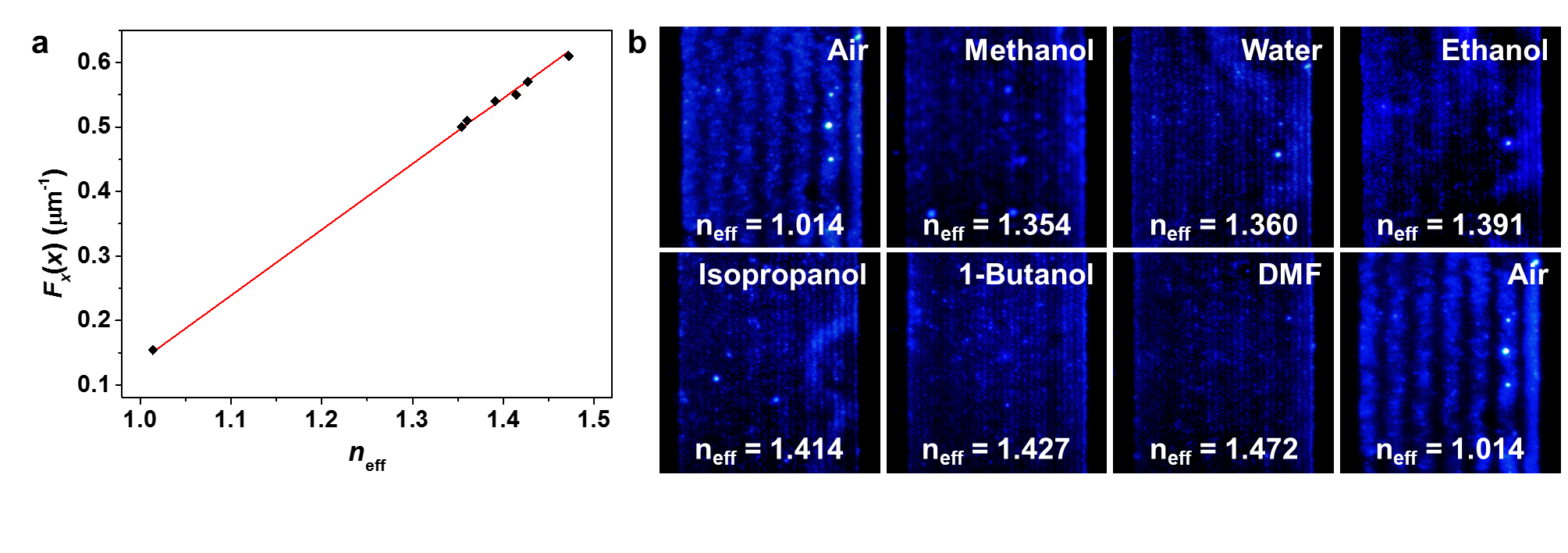
**

**Fig. S10. Response to different solvents.** (a) Interference fringe (*x*-direction) frequency versus effective index (*n*_eff_) curve for different surrounding media (air, methanol, water, ethanol, isopropanol, 1-butanol, and DMF). (b) PL images for the interference fringes supported by a 50-µm-wide Au stripe immersed in different dielectrics. The period for the fringes decreases with increasing the effective index by changing to solvents with higher dielectric constant and recovers after the solvent is evaporated by N_2_ flow. The free-space wavelength is 980 nm and the incident angle is 60°.

**
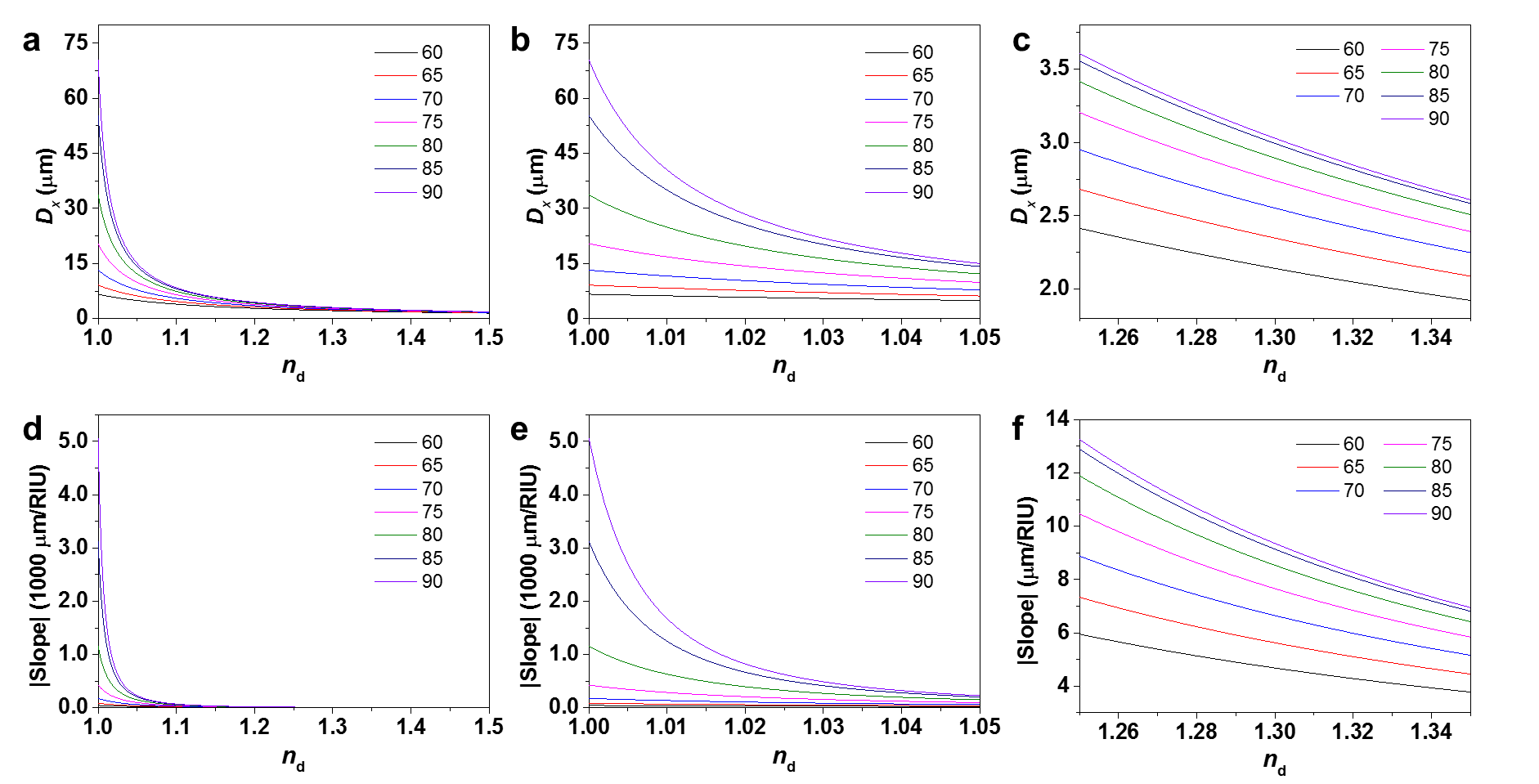
**

**Fig. S11. Calculated *D_x_*–*n*_d_ relationship at different incident angles.** (a–c) *D_x_*–*n*_d_ curves at different incident angles. (d–f) The absolute value of the slope for the *D_x_*–*n*_d_ curve (i.e. refractive index sensitivity) is plotted for different incident angles, showing that the refractive index sensitivity increases dramatically with the increase of the incident angle.

**
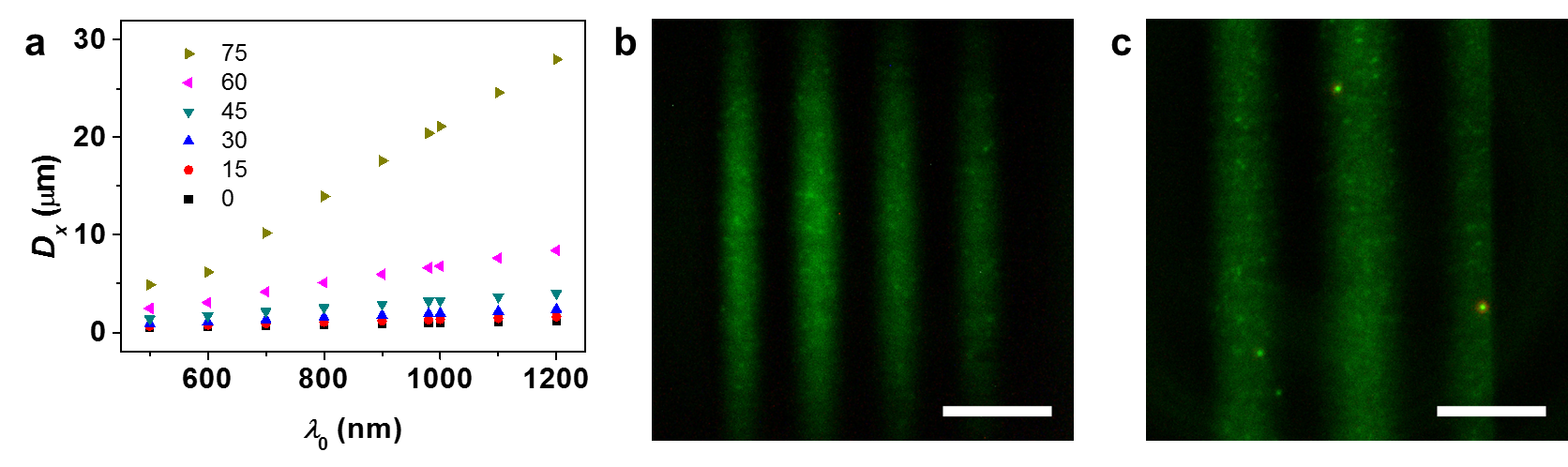
**

**Fig. S12. Influence of the incident light wavelength on the periods of the interference fringes.** (a) Calculated *d*-spacing (period) values of the *x*-direction interference fringes (if present) versus free-space wavelength curves for different incident angles, showing that the *d*-spacing will increase at all incident angles when the incident free-space wavelength is increased. (b,c) Interference fringes supported by a 50-µm-wide Au stripe excited by a (b) 808 nm or (c) 980 nm laser with the same incident angle (~75°), confirming the calculation results shown in panel (a). Another type of UCNPs, *β*–NaGdF_4_:Yb,Er@NaGdF_4_:Nd NPs, which can be excited by either 808 or 980 nm laser[^1^](#_ENREF_1), were used to probe the intensity dispersion of the interference fringes under incident light with the free-space wavelength of 808 or 980 nm, respectively. Scale bar: 25 µm.

**
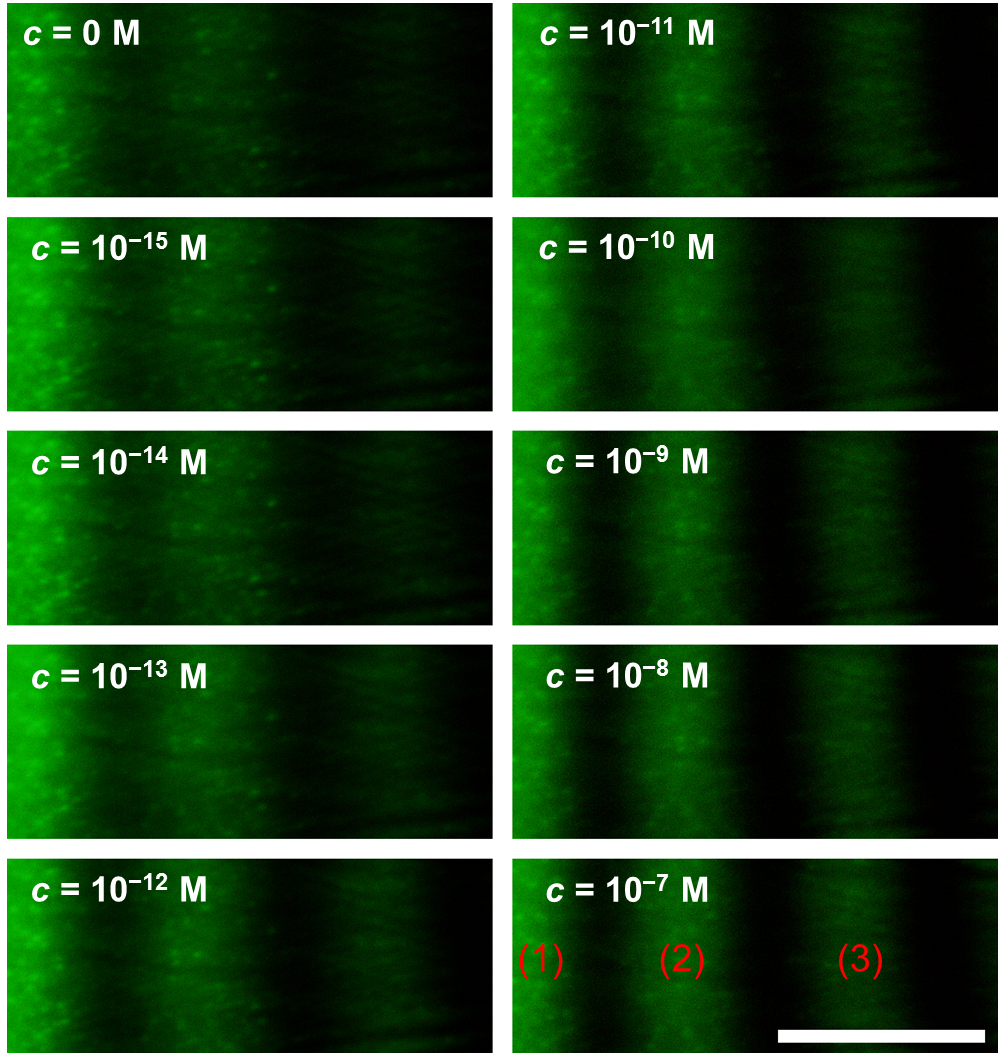
**

**Fig. S13. Real-colour image of the interference fringes showing the response to streptavidin for biotinylated Au–UCNP sensors.** The fringes supported on the biotinylated Au–UCNP pattern show a significant systematic shift (decrease of *d*-spacing) after the binding of streptavidin by surface biotin groups. For the sensing of streptavidin at each bulk concentration, after the binding of surface biotin and streptavidin in the microfluidic channel (illustrated in Fig. S14), the Au–UCNP pattern and the channel is rinsed with DI water for three times to remove excessive unspecifically bound streptavidin molecules and dried under N_2_ flow. PL images were collected to verify that stable fringes were obtained for the calculation of spatial periods. The three bright stripes highlighted correspond to the three intensity peaks shown in Figure 5a in the main text. Scale bar: 50 µm.

**
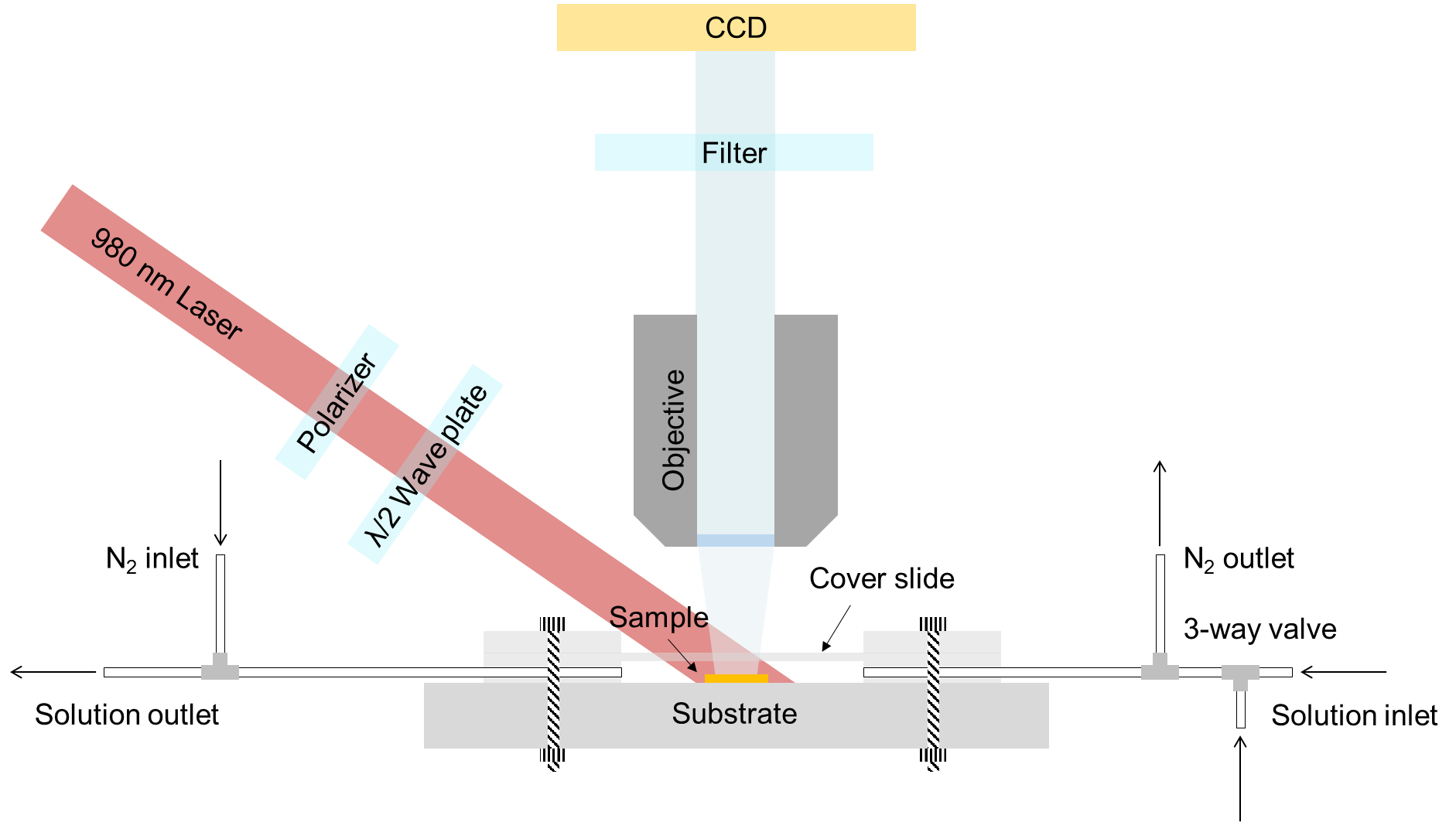
**

**Fig. S14. Schematic illustration for the microfluidic channels used in the solvent and streptavidin sensing experiments.** The biotinylated Au-UCNP pattern supported on a SiO_2_/Si substrate is placed in a PDMS channel with a volume of about 10 µL, which is sealed with a cover slide (0.15 mm in thickness) and tightened with clamps. The sample is fixed on the specimen holder of the optical microscope. A 980 nm laser is obliquely shed onto the sample and the upconversion emission is collected by the objective. Different solvents or solutions with different streptavidin concentrations were injected by syringe pumps. N_2_ flow was used to blow dry the samples before changing different solvents.


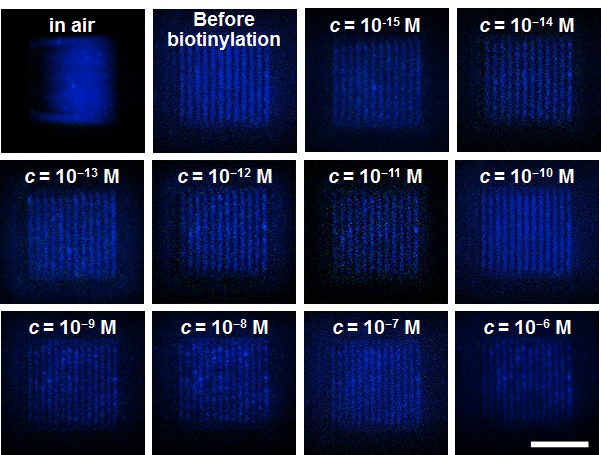


**Fig. S15. Response to streptavidin solution of different concentrations for biotinylated Au-UCNP sensors.** Directly observed interference fringes supported on the Au-UCNP square immersed in air or aqueous solution of streptavidin with different concentrations. Scale bar: 20 µm.

**
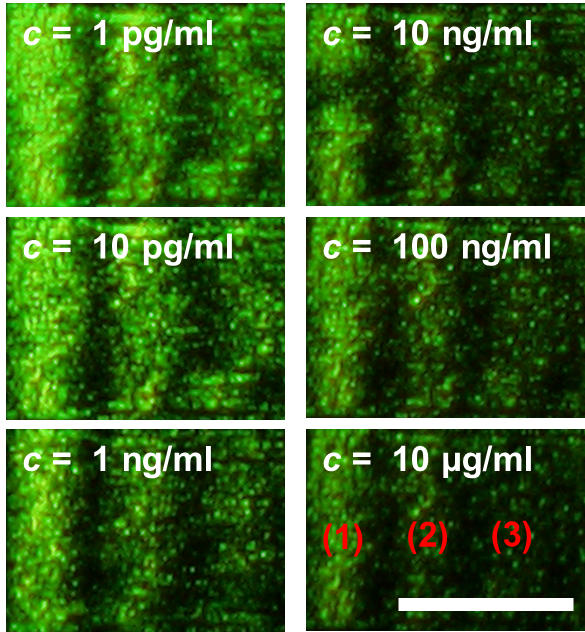
**

**Fig. S16. Real-colour image of the interference fringes showing the response to PSA for biotinylated Au–UCNP sensors.** The fringes supported on the antibody-modified Au–UCNP pattern show a significant systematic shift (decrease of *d*-spacing) after the binding of PSA by surface antibody groups. For the sensing of PSA at each bulk concentration, after the binding of surface antibody and PSA in the microfluidic channel (illustrated in Fig. S13), the Au–UCNP pattern and the channel are rinsed with DI water for three times to remove excessive non-specifically bound PSA molecules and dried under N_2_ flow. PL images were collected to verify that stable fringes were obtained for the calculation of spatial periods. The three bright stripes highlighted correspond to the three intensity peaks shown in Figure 5e in the main text. Scale bar: 100 µm.

**Materials and Methods**

**Chemicals.** Sodium trifluoroacetate (98%, Sigma-Aldrich), yttrium trifluoroacetate (99.99%, Sigma-Aldrich), ytterbium(III) oxide (Yb_2_O_3_, 99.9%, Sigma-Aldrich), thulium oxide (Tm_2_O_3_, 99.9%, Sigma-Aldrich), erbium oxide (Er_2_O_3_, 99.9%, Sigma-Aldrich), trifluoroacetic acid (99%, Sigma-Aldrich), oleic acid (90%, Sigma-Aldrich), oleylamine (70%, Sigma-Aldrich), 1-octadecene (90%, Sigma-Aldrich), (+)-biotin N-hydroxysuccinimide ester (98%, Sigma-Aldrich), streptavidin from *Streptomyces avidinii* (Sigma-Aldrich), ethanethiol (97%, Sigma-Aldrich), and cysteamine (98%, Sigma-Aldrich) were all used as received without further purification. The deionized water used in the experiment is ultra-pure (MilliQ, 18 MΩ). Yb(CF_3_COO)_3_, Tm(CF_3_COO)_3_ or Er(CF_3_COO)_3_ powders were prepared by the reaction between Yb_2_O_3_, Tm_2_O_3_, or Er_2_O_3_ and trifluroacetic acid in aqueous solutions under reflux. The product was filtered to remove any solid impurities and subsequently dried in a vacuum oven at 120 °C overnight.

**Synthesis of *β*–NaYF_4_:Yb,Tm NPs.** *β*–NaYF_4_:Yb,Tm NPs were prepared with a previously reported two-step high-temperature thermolysis method[^2^](#_ENREF_2)^,^[^3^](#_ENREF_3). In the first step, CF_3_COONa (1 mmol), Y(CF_3_COO)_3_ (0.795 mmol), Yb(CF_3_COO)_3_ (0.20 mmol), and Tm(CF_3_COO)_3_ (0.005 mmol) were mixed with oleic acid (10 mmol), oleylamine (10 mmol), and 1-octadecene (20 mmol) in a 50 mL three-necked flask, degassed and heated to 120 °C for 30 min to remove the low-boiling-point impurities. The solution is subsequently heated to 300 °C under a N_2_ atmosphere at the rate of 20 °C/min and then kept for 30 min. After the resultant solution was cooled down to room temperature by air, excessive ethanol was added into the flask to precipitate the as-obtained *α*–NaYF_4_:Yb,Tm NPs. The precipitates were washed with cyclohexane and ethanol and centrifuged for three times and then collected. In the second step, the as-prepared α-NaYF_4_:Yb,Tm NPs and 1 mmol of CF_3_COONa were mixed with OA (20 mmol) and 1-octadecene (20 mmol) in a 50 mL three-necked flask. The degassing and high-temperature reaction procedure are similar to that in the first step, except that the final reaction temperature is raised to 320 °C. The as-obtained *β*–NaYF_4_:Yb,Tm NPs were centrifuged and washed by toluene and ethanol for three times. The final products were collected and redispersed in toluene before characterization and applications. *β*–NaYF_4_:Yb,Er NPs were prepared with similar method.

**Fabrication of Au patterns.** Au patterns were fabricated on SiO_2_/Si substrates using standard photolithography or electron-beam lithography method. The thickness of the Au patterns is 75 nm. The quality of the Au patterns was verified by optical, SEM images and AFM profiles.

**Deposition of the *β*–NaYF_4_:Yb,Tm NPs onto Au patterns.** Toluene dispersion of *β*–NaYF_4_:Yb,Tm NPs was deposited onto the substrates by spin-coating methods. In a typical spin-coating process, 20 µL of diluted dispersion of *β*–NaYF_4_:Yb,Tm NPs were applied onto the substrates with a micropipette, subsequently rotated at 8000 rpm, and then dried under a N_2_ flow. The density of NPs on Au patterns, which is examined by PL image, SEM, and AFM, can be tuned by the concentration of the NP dispersions or the spin coating speed.

**Upconversion PL measurement for *β*–NaYF_4_:Yb,Tm NPs.** The upconversion PL images and spectra of the as-prepared *β*–NaYF_4_:Yb,Tm NPs were recorded by a homebuilt microscope/spectrometer with a continues wave diode laser (maximum 1.5 W at 980 nm). An Olympus BX51 optical microscope (with 10 ×, NA = 0.25; 50 ×, NA = 0.50; and 100 ×, NA = 0.8 LMPLFLN-BD objectives) was integrated with a colour CCD camera (Olympus, DP73), and a monochromator (Acton SpectraPro 2300i) with a liquid-nitrogen-cooled CCD camera (Princeton Instruments Spec10). A short-pass filter (700 nm cut-on, Newport) and other proper band-pass filters were used during the imaging/spectra acquisitions.

**Other characterization.** Transmission electron microscopy (TEM) images were acquired on an FEI T12 (120 kV). SEM images were obtained on a Zeiss Supra 40VP SEM. AFM profiles were performed on a Bruker Dimension Icon Scanning Probe Microscope in tapping mode.

**Numerical simulation on the interference fringes by the RWP and SPPs.** The complex dielectric constants are from Rakic and co-workers’ report[^4^](#_ENREF_4). The schematic illustration for the interference between the RWP and SPPs[^5^](#_ENREF_5) is shown in Figure 2c in the main text. In a simplified model, assuming that the SPPs are not attenuated and the initial intensity for the SPPs is equal to the RWP, we can derive that

$$A_{Sx}(t)=A_{0}\sin\left( k_{Sx}x+\omega_{0}t+\varphi_{0} \right)$$

$$A_{R}(t)=A_{0}\sin\left( k_{R}x+\omega_{0}t+\varphi_{0} \right)$$

The interference between them forms a new wave

$${A_{x}\left( t \right)=A}_{Sx}(t)+A_{R}(t) =A_{0}\left[ \sin\left( k_{Sx}x+\omega_{0}t+\varphi_{0} \right)+\sin\left( k_{R}x+\omega_{0}t+\varphi_{0} \right) \right]$$

$A_{x}(t)={2A}_{0}\left[ \sin\left( \frac{k_{Sx}+k_{R}}{2}x+\omega_{0}t+\varphi_{0} \right)\cos\left( \frac{k_{Sx}-k_{R}}{2}x \right) \right]$.

The intensity dispersion is

$$I_{x}(t)=4A_{0}^{2}\left[ \sin\left( \frac{k_{Sx}+k_{R}}{2}x+\omega_{0}t \right)\cos\left( \frac{k_{Sx}-k_{R}}{2}x \right) \right]^{2}$$

$I_{x}(t)=I_{x0}\left\{ 1-cos \left[ \left( k_{Sx}+k_{R} \right)x+{2\omega}_{0}t+2\varphi_{0} \right] \right\}\left\{ 1+cos \left[ \left( k_{Sx}-k_{R} \right)x \right] \right\}$.

For $k_{Sx}=k_{0}n_{\mathrm{eff}}$ and $k_{R}=k_{0}\sin\theta$, we can assume that $k_{Sx}+k_{R}\gg k_{Sx}-k_{R}$ and thus define the $\left\{ 1-cos \left[ \left( k_{Sx}+k_{R} \right)x+{2\omega}_{0}t+2\varphi_{0} \right] \right\}$ term as the high-frequency part and the $\left\{ 1+cos \left[ \left( k_{Sx}-k_{R} \right)x \right] \right\}$ term as the low-frequency part. As shown in Figure S17, the time-dependent high-frequency part is enveloped by the time-independent low-frequency curve. Considering the very high angular frequency for the incident light, the high-frequency vibration cannot be distinguished by conventional optical acquisitions with long integration time. As a result, the observed intensity dispersion should follow the upper envelope curve of the high-frequency vibrations, that is, the low-frequency term.


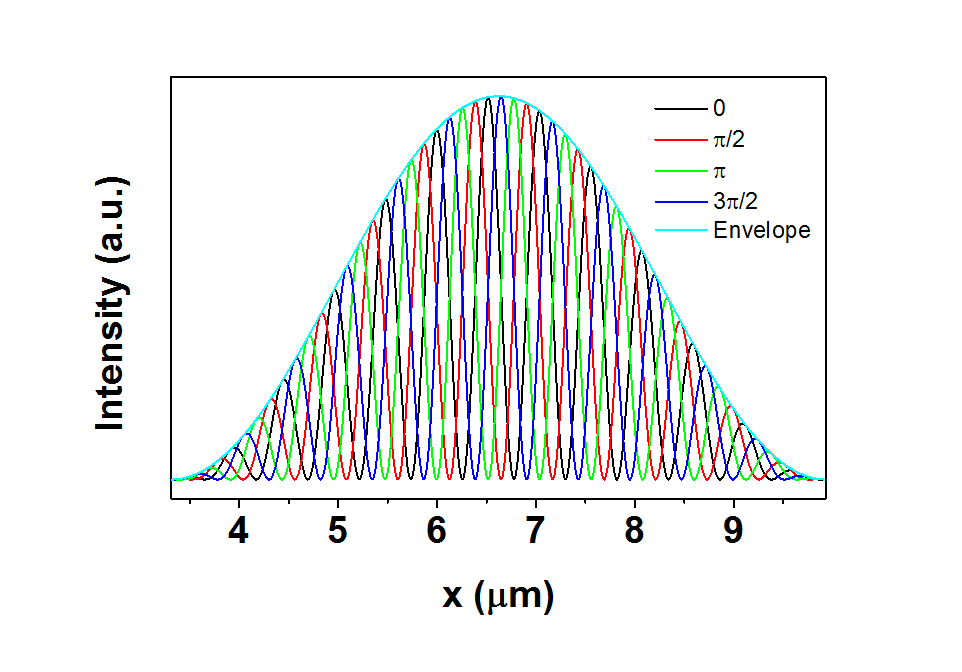


**Fig. S17.** Time-dependent intensity dispersion for the interference between the RWP and SPPs in one period, showing the envelope curve (low-frequency, cyan) and the time-dependent intensity curves (high-frequency) for 2*ω*_0_*t* = 0 (black), π/2 (red), π (green) and 3π/2 (blue), respectively. The incident light beam (*λ*_0_ = 980 nm) is obliquely shed onto the surface at *θ* = 60°.

Therefore, the intensity dispersion for the interference between the RWP and SPPs is time-independent, which is

$$I_{x}=2I_{0}\left\{ 1+cos \left[ \left( k_{Sx}-k_{R} \right)x \right] \right\}$$

$$I_{x}=2I_{x0}\left\{ 1+cos \left[ k_{0}\left( n_{\mathrm{eff}}-\sin\theta\right)x \right] \right\}=2I_{x0}\left\{ 1+cos \left[ \frac{2\pi}{\lambda_{0}}\left( n_{\mathrm{eff}}-\sin\theta\right)x \right] \right\}$$

Then, the *d*-spacing (period) and frequency of the interference fringes along the *x* direction is defined by

$$D_{x}=\frac{\lambda_{0}}{n_{\mathrm{eff}}-\sin\theta}$$

$$F_{x}=\frac{n_{\mathrm{eff}}-\sin\theta}{\lambda_{0}}$$

The period and frequency of the interference fringes in this simplified model share the same expressions with the one that takes the attenuation effects and the excitation efficiency of the SPPs into account because both the attenuation and the efficiency terms do not affect the period but just the intensity of the interference fringes. Similarly, we can derive the intensity dispersion equations for the interference between the RWP and SPPs with both the attenuation effect and excitation efficiency taken into account. The numerical calculations, as shown in the main text, fit the experiment results precisely.

In *y*-direction, two SPPs traveling in opposite directions (denoted as S*y*+ and S*y*−) can be excited and the interference of these two SPPs will result in a standing wave since they share the same frequency (*ω* = *ω*_0_).

The two traveling waves can be represented by

$$A_{Sy+}(t)=A_{Sy,0}\sin\left( k_{Sy}y+\omega t \right)$$

$$A_{Sy-}(t)=A_{Sy,0}\sin\left( k_{Sy}y-\omega t \right)$$

And the interfered wave will be

$$A_{Sy}(t)=A_{Sy+}(t)+A_{Sy-}(t)=A_{Sy,0}\left[ \sin\left( k_{Sy}y+\omega t \right)+\sin\left( k_{Sy}y-\omega t \right) \right]$$

$$A_{Sy}(t)=2A_{Sy,0}\sin\left( k_{Sy}y \right)\cos\left( \omega t \right)$$

Therefore, the intensity distribution along *y*-direction is

$$I_{y}(t)=4A_{Sy,0}^{2}\left[ \sin\left( k_{Sy}y \right)\cos\left( \omega t \right) \right]^{2}$$

$$I_{y}(t)=I_{Sy,0}\left[ 1-\cos\left( 2k_{Sy}y \right) \right]\left[ 1-\sin\left( 2\omega t \right) \right]$$

$$I_{y}\leq2I_{Sy,0}\left[ 1-\cos\left( 2k_{S,y}y \right) \right]$$

And thus, we have the intensity dispersion for the lateral fringes dispersed along the *y*-direction.

**Conversion from the UCPL intensity to local field intensity.** The UCPL emissions of RE-doped UCNPs involve multiple-photon transitions, and the emission intensity (*I*_UC_) versus excitation power density (*P*_E_) relationship follows the equation of $I\propto P^{n}$, when *n* is the number of photons involved to excite the activator ion from ground state to specific excited state^2,3^. For example, the 476 nm emission from *β*–NaYF_4_:Yb,Tm NPs is corresponded to the three-photon transition (^1^G_4_ to ^3^H_6_)^3^, and the emission intensity $I_{476}\propto I_{\mathrm{LF}}^{3}$ (Fig. S18a), where *I*_LF_ is the local field intensity at the position of the *β*–NaYF_4_:Yb,Tm NPs. Therefore, the local field intensity $I_{\mathrm{LF}}\propto\sqrt[3]{I_{476}}$, and the relative intensity dispersion of the local field intensity can be converted from the measured upconversion emission intensity (Fig. S18b).


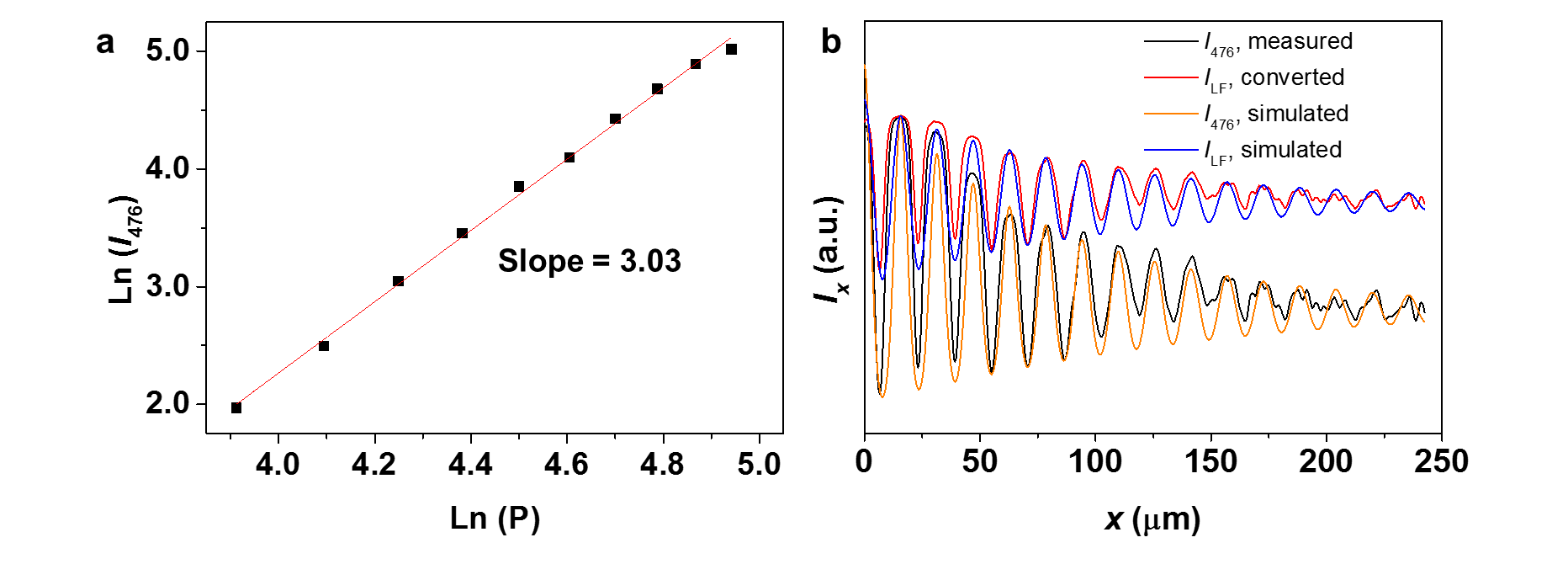


**Fig. S18.** **Conversion from UCPL intensity to local field intensity.** (a) Emission intensity versus excitation power density curves for the 476 nm emission of *β*–NaYF_4_:Yb,Tm NPs when excited by a 980 nm CW laser. The slope for the Ln(*I*_476_)–Ln(*P*) curve is 3.03. (b) Measured UCPL intensity (476 nm) dispersion, local field intensity converted based on the $I_{\mathrm{LF}}\propto\sqrt[3]{I_{476}}$ relationship, and simulated intensity dispersion curves.

**Effects of dielectric environment on the interference between the RWP and SPPs.** The period and frequency for the interference fringes, defined by Equations 4 and 5 in the main text, is determined by the effective index when the free-space wavelength and the incident angle is fixed. And, since $n_{\mathrm{eff}}=\sqrt{\frac{{\varepsilon_{d}\varepsilon}_{\mathrm{Au}}}{\varepsilon_{d}+\varepsilon_{\mathrm{Au}}}}$ is dependent on the dielectric constant of the surrounding dielectrics, interference fringe patterns with different periods should be observed when the Au patterns are immersed in different solvents, including water, methanol, etc. The calculated results are listed in Tables S1.

**Table S1. Calculated period of the interference fringe patterns in different solvents**

| **Solvents** | ***n*_d_** | ***ε*_d_** | ***n*_eff_** | ***D_x_* (µm)** | | | | | | |
| --- | --- | --- | --- | --- | --- | --- | --- | --- | --- | --- |
|  |  |  |  | **θ = 0°** | **15°** | **30°** | **45°** | **60°** | **75°** | **90°** |
| Air | 1.000 | 1.000 | 1.014 | 0.97 | 1.30 | 1.91 | 3.19 | 6.63 | 20.4 | 70.5 |
| Methanol | 1.321 | 1.746 | 1.354 | 0.72 | 0.89 | 1.15 | 1.52 | 2.01 | 2.53 | 2.77 |
| Water | 1.327 | 1.761 | 1.360 | 0.72 | 0.89 | 1.14 | 1.50 | 1.98 | 2.49 | 2.72 |
| Ethanol | 1.355 | 1.837 | 1.391 | 0.70 | 0.87 | 1.10 | 1.43 | 1.87 | 2.31 | 2.51 |
| Isopropanol | 1.377 | 1.896 | 1.414 | 0.69 | 0.85 | 1.07 | 1.39 | 1.79 | 2.19 | 2.37 |
| 1-Butanol | 1.389 | 1.929 | 1.427 | 0.69 | 0.84 | 1.06 | 1.36 | 1.75 | 2.13 | 2.30 |
| DMF | 1.430 | 2.045 | 1.472 | 0.67 | 0.81 | 1.01 | 1.28 | 1.62 | 1.94 | 2.08 |

As shown in Figure S10, the experimentally observed frequency values for different dielectric environments match well with the theoretically calculated results, showing an increment in the frequency with the increase in the refractive index in the surrounding dielectrics.

**Effects of free-space wavelength on the interference between the RWP and SPPs.** According to Equation 4 in the main text, the period for the interference fringes will increase with the increase of free-space wavelength. In addition, the effective index is also dependent on the free-space wavelength because the complex dielectric constant of Au varies from the visible to NIR regime. The effective index and corresponded interference fringe periods (if present) for different free-space wavelength is listed in Table S2.

**Table S2. Calculated period of the interference fringe patterns on air/Au surface with different incident wavelengths and angles**

| ***λ*_0_ (nm)** | ***n*_eff_** | ***Dx* (µm)** | | | | | | |
| --- | --- | --- | --- | --- | --- | --- | --- | --- |
|  |  | ***θ* = 0°** | **15°** | **30°** | **45°** | **60°** | **75°** | **90°** |
| 500 | 1.068 | 0.47 | 0.62 | 0.88 | 1.38 | 2.47 | 4.88 | 7.31 |
| 600 | 1.063 | 0.56 | 0.75 | 1.07 | 1.69 | 3.05 | 6.18 | 9.53 |
| 700 | 1.035 | 0.68 | 0.90 | 1.31 | 2.14 | 4.15 | 10.18 | 20.17 |
| 800 | 1.023 | 0.78 | 1.05 | 1.53 | 2.53 | 5.09 | 13.95 | 34.40 |
| 900 | 1.017 | 0.88 | 1.19 | 1.74 | 2.90 | 5.96 | 17.59 | 52.64 |
| 980 | 1.014 | 0.97 | 1.30 | 1.91 | 3.19 | 6.63 | 20.42 | 70.46 |
| 1000 | 1.014 | 0.99 | 1.33 | 1.95 | 3.27 | 6.79 | 21.12 | 75.39 |
| 1100 | 1.011 | 1.09 | 1.46 | 2.15 | 3.62 | 7.61 | 24.59 | 103.13 |
| 1200 | 1.009 | 1.19 | 1.60 | 2.36 | 3.98 | 8.40 | 27.99 | 136.33 |

As shown in Table S2 and Figure S11, the period of the interference fringes (if present) all increase with the wavelength increase for various incident angles.

**Surface modification of the Au patterns with biotin and the response to streptavidin.** The biotinylated Au patterns were prepared used a modified method which was reported previously[^6^](#_ENREF_6)^,^[^7^](#_ENREF_7). The Au patterns with randomly dispersed UCNPs on the surface (Fig. S2) were first immersed in a solution of cysteamine (2 mM, 24h) to form a self-assembled monolayer (SAM)[^8^](#_ENREF_8) resulting in a surface coverage of amine binding sites. The patterns were subsequently washed with water to remove the excessive cysteamine solution. Next, biotin was covalently attached to the amino groups by immersing the patterns in (+)-biotin N-hydroxysuccinimide ester (2 mg/mL) in dry DMF solution for 30 min. The patterns were then rinsed with water to remove excessive (+)-biotin N-hydroxysuccinimide ester solutions before use. For the ultrasensitive detection of streptavidin, the Au pattern and the channel is rinsed with DI water for to remove excessive unspecifically bound streptavidin molecules and dried under N_2_ flow after the binding of surface biotin and streptavidin in the microfluidic channel (illustrated in Fig. S14). PL images were collected to verify that stable fringes were obtained for the calculation of spatial periods. For the *in situ* detection of streptavidin in solutions, PL images were captured when the Au pattern is immersed in streptavidin solutions until stable interference fringes were obtained to calculate the spatial period for the interference fringes for streptavidin solutions with different bulk concentrations.

**Table S3. Comparison of different PSA detection methods**

| **Materials** | **Limit of Detection (LOD)** | **Detection Method** | **Label-free** | **Further Amplified** | **Remarks** | **Ref.** |
| --- | --- | --- | --- | --- | --- | --- |
| **Au-UCNP** | **1 pg/ml (30 fM)** | **Optical image Spatial measurement** | **Yes** | **No** | **Low cost Easy processing Spectrometer-free** | **This work** |
| Si nanowire arrays | 0.9 pg/ml | Field-effect devices Electrical signal | Yes | No | High cost Complicated processing | 9 |
| Silicon nitride microcantilever | 0.2 ng/ml | Microcantilever frequency | Yes | No | High cost Complicated processing | 10 |
| NP-based bio-barcode assay | 10 fM | Scanometric detection Intensity | Yes | Yes (DNA) | High cost Complicated processing | 11 |
| NP-based bio-barcode assay | 30 aM | Scanometric detection Intensity | Yes | Yes (DNA) | High cost Complicated processing | 12 |
| Single molecule ELISA | 0.4 fM | Fluorescence Spectrum | No | Yes (enzyme) | ELISA Complicated processing | 13 |
| Microcantilever & plasmonic | 0.1 pg/ml | Microcantilever frequency Scattering spectrum | Yes | No | Sandwich immunoassay High cost Complicated processing | 14 |
| Plasmonic ELISA | 10^-18^ g/ml | Colorimetric method | No | Yes (enzyme) | ELISA Complicated processing Small detection range, low-concentration only | 15 |
| Au and Ag NPs | 10^-20^ M | NP assembly Circular dichroism absorption | Yes | No | Sandwich immunoassay Advanced instrumentation | 16 |
| Au NP and NRs | 20 fM | NP assembly Dynamic Light Scattering | Yes | No | Sandwich immunoassay Advanced instrumentation | 17 |
| Au nanostars Enzyme-guided growth | 10^-18^ g/ml | Scattering spectra | Yes | Yes (enzyme) | Small detection range, low-concentration only | 18 |

**References**

1. Y. F. Wang, *et al.* Nd^3+^-Sensitized upconversion nanophosphors: efficient *in vivo* bioimaging probes with minimized heating effect. *ACS Nano* **7**, 7200-7206, (2013).

2. H. X. Mai,  *et al.* High-quality sodium rare-earth fluoride nanocrystals: controlled synthesis and optical properties. *J. Am. Chem. Soc.* **128**, 6426-6436 (2006).

3. A. X. Yin, Y. W. Zhang, L. D. Sun, C. H. Yan, Colloidal synthesis and blue based multicolor upconversion emissions of size and composition controlled monodisperse hexagonal NaYF_4_:Yb,Tm nanocrystals. *Nanoscale* **2**, 953-959 (2010).

4. A. D. Rakic, A. B. Djurisic, J. M. Elazar, M. L. Majewski, Optical properties of metallic films for vertical-cavity optoelectronic devices. *Appl. Opt.* **37**, 5271-5283 (1998).

5. P. Genevet, *et al.* Controlled steering of Cherenkov surface plasmon wakes with a one-dimensional metamaterial. *Nature Nanotechnol.* **10**, 804-809 (2015).

6. A. J. Haes, R. P. Van Duyne, A nanoscale optical biosensor: sensitivity and selectivity of an approach based on the localized surface plasmon resonance spectroscopy of triangular silver nanoparticles. *J. Am. Chem. Soc.* **124**, 10596-10604 (2002).

7. Y. J. Tang, R. Mernaugh, X. Q. Zeng, Nonregeneration protocol for surface plasmon resonance: Study of high-affinity interaction with high-density biosensors. *Anal. Chem.* **78**, 1841-1848 (2006).

8. C. D. Bain, G. M. Whitesides, Formation of two-component surfaces by the spontaneous assembly of monolayers on gold from solutions containing mixtures of organic thiols. *J. Am. Chem. Soc.* **110**, 6560-6561 (1988).

9. G. F. Zheng, F. Patolsky, Y. Cui, W. Wang, C. M. Lieber, Multiplexed electrical detection of cancer markers with nanowire sensor arrays. *Nature Biotechnol.* **23**, 1294-1301 (2005).

10. G. H. Wu, *et al.* Bioassay of prostate-specific antigen (PSA) using microcantilevers. *Nature Biotechnol.* **19**, 856-860 (2001).

11. C. S. Thaxton, *et al.* Nanoparticle-based bio-barcode assay redefines "undetectable'' PSA and biochemical recurrence after radical prostatectomy. *Proc. Natl. Acad. Sci. USA* **106**, 18437-18442 (2009).

12. J. M. Nam, C. S. Thaxton, C. A. Mirkin, Nanoparticle-based bio-bar codes for the ultrasensitive detection of proteins. *Science* **301**, 1884-1886 (2003).

13. D. M. Rissin, *et al.* Single-molecule enzyme-linked immunosorbent assay detects serum proteins at subfemtomolar concentrations. *Nature Biotechnol.* **28**, 595-599 (2010).

14. P. M. Kosaka, *et al.* Detection of cancer biomarkers in serum using a hybrid mechanical and optoplasmonic nanosensor. *Nature Nanotech.* **9**, 1047-1053 (2014).

15. R. de la Rica, M. M. Stevens, Plasmonic ELISA for the ultrasensitive detection of disease biomarkers with the naked eye. *Nature Nanotech.* **7**, 821-824 (2012).

16. X. L. Wu, *et al.* Unexpected chirality of nanoparticle dimers and ultrasensitive chiroplasmonic bioanalysis. *J. Am. Chem. Soc.* **135**, 18629-18636 (2013).

17. X. Liu, *et al.* A one-step homogeneous immunoassay for cancer biomarker detection using gold nanoparticle probes coupled with dynamic light scattering. *J. Am. Chem. Soc.* **130**, 2780-2782 (2008).

18. L. Rodriguez-Lorenzo, R. de la Rica, R. A. Alvarez-Puebla, L. M. Liz-Marzan, M. M. Stevens, Plasmonic nanosensors with inverse sensitivity by means of enzyme-guided crystal growth. *Nature Mater.* **11**, 604-607 (2012).
